# SARS-CoV-2 vaccine protection and deaths among US veterans during 2021

**DOI:** 10.1126/science.abm0620

**Published:** 2021-11-04

**Authors:** Barbara A. Cohn, Piera M. Cirillo, Caitlin C. Murphy, Nickilou Y. Krigbaum, Arthur W. Wallace

**Affiliations:** 1 Public Health Institute, Oakland, CA, USA.; 2 San Francisco Veterans Affairs Medical Center, San Francisco, CA, USA.; 3 School of Public Health, University of Texas Health Science Center at Houston, Houston, TX, USA.; 4 Department of Anesthesiology and Perioperative Care, University of California, San Francisco, San Francisco, CA, USA.

## Abstract

We report severe acute respiratory syndrome coronavirus 2 (SARS-CoV-2) vaccine effectiveness against infection (VE-I) and death (VE-D) by vaccine type in 780,225 veterans in the Veterans Health Administration, covering 2.7% of the US population. From February to October 2021, VE-I declined for all vaccine types, and the decline was greatest for the Janssen vaccine, resulting in a VE-I of 13.1%. Although breakthrough infection increased risk of death, vaccination remained protective against death in persons who became infected during the Delta variant surge. From July to October 2021, VE-D for age <65 years was 73.0% for Janssen, 81.5% for Moderna, and 84.3% for Pfizer-BioNTech; VE-D for age ≥65 years was 52.2% for Janssen, 75.5% for Moderna, and 70.1% for Pfizer-BioNTech. Findings support continued efforts to increase vaccination, booster campaigns, and multiple additional layers of protection against infection.

## Veterans’ virus affairs

The US Department of Veterans Affairs (VA) provides life-long healthcare to military personnel, and VA data offer a unique resource for public health surveillance in the United States. Cohn *et al*. examined data from over 780,000 individuals to track the effects of vaccination rollout as the Delta variant of severe acute respiratory syndrome coronavirus 2 (SARS-CoV-2) emerged in the United States between February and September of 2021. Although messenger RNA (mRNA) and viral vector vaccines have effectively prevented clinically significant disease, SARS-CoV-2 Delta transmission has surged. Vaccine breakthrough infections have predominated in those given the viral vector vaccine but also occur among those given mRNA vaccines. By September 2021, the protection offered by the viral vector vaccine during the study declined to about 13% against infection and to about 50% against death. Unvaccinated individuals remained at the highest risk of infection, severe disease, and death. —CA

## Teaser

Vaccine protection against SARS-CoV-2 infection declined through August 2021 as the Delta variant emerged in the United States.

The mRNA vaccines BNT162b2 (Pfizer-BioNTech) and mRNA-1273 (Moderna) and the viral vector vaccine JNJ-78436735 (Janssen) have effectively prevented clinically recognized disease caused by severe acute respiratory syndrome coronavirus 2 (SARS-CoV-2) since their rollout in the United States in late 2020 (*1*, *2*). Vaccines have also reduced the incidence of asymptomatic infection and associated infectivity (*3*). However, by July 2021, the United States experienced a surge in cases of COVID-19, dominated by the B.1.617.2 (Delta) variant (*4*, *5*). Initial reports, including follow-up of the Pfizer-BioNTech and Moderna trials (*6*–*8*), suggested sustained vaccine protection (*9*), but three reports by the US Centers for Disease Control and Prevention (CDC) in August 2021 (*10*–*12*) demonstrated that protection against infection had declined in mid-summer as the Delta variant rose to dominance; protection against hospitalization and death remained high (*13*–*15*). Breakthrough infections, illness, hospitalizations, and deaths have since continued to emerge in vaccine recipients.

This phenomenon has been most comprehensively monitored in Israel, where high levels of transmission of the Delta variant led to a resurgent outbreak in mid-June 2021 (*16*) despite a successful nationwide campaign to vaccinate the population (*17*). Israel authorized boosters of the Pfizer-BioNTech vaccine for adults age ≥60 years in July 2021 and extended this authorization to adults age ≥50 years in August 2021 (*18*). Rates of infection and severe illness subsequently declined in those who received a booster (*19*). Largely on the basis of these data, as well as data from the UK (*20*, *21*), the US Food and Drug Administration (FDA) authorized boosters of the Pfizer-BioNTech vaccine for older (age ≥65 years) and higher-risk adults in September 2021 (*22*); the FDA similarly authorized boosters of the Moderna vaccine in October 2021, as well as boosters for all recipients of the Janssen vaccine (*23*).

The debate over boosters in the United States (*24*) has laid bare the limitations of its public health infrastructure: National data on vaccine breakthrough are inadequate. The CDC transitioned in May 2021 from monitoring all breakthrough infections to focus on identifying and investigating only hospitalized or fatal cases attributable to any cause, including causes not related to COVID-19 (*25*). Some data on vaccinations, infections, and deaths are collected through a patchwork of local health departments (*10*), but these data are frequently out of date and difficult to aggregate at the national level. We addressed this gap and examined SARS-CoV-2 infection and deaths by vaccination status in 780,225 veterans during the period 1 February 2021 to 1 October 2021, encompassing the emergence and dominance of the Delta variant in the United States.

The distribution of SARS-CoV-2 infection by demographics, comorbidity, and vaccination status is shown in table S1 for 1 February 2021 to 1 October 2021 (*n* = 780,225 subjects). The percentage of polymerase chain reaction (PCR) test positivity is higher in veterans who were unvaccinated (25.8%), female (15.8%), Hispanic (13.9%), American Indian/Alaska Native (14.7%) or Native Hawaiian/Pacific Islander (14.2%), age <50 years at time of reverse transcription PCR (RT-PCR) assay (19.1%), and had a lower comorbidity score (16.2% for Charlson Comorbidity Index = 0) (*26*); 33,514 positive PCR tests occurred in 498,148 fully vaccinated veterans. The distribution of vaccine type by demographic is shown in table S2. Vaccine type differed by age: Younger (age <50 years) veterans were more likely to have received the Janssen vaccine than either Moderna or Pfizer-BioNTech.

**Fig. 1.**
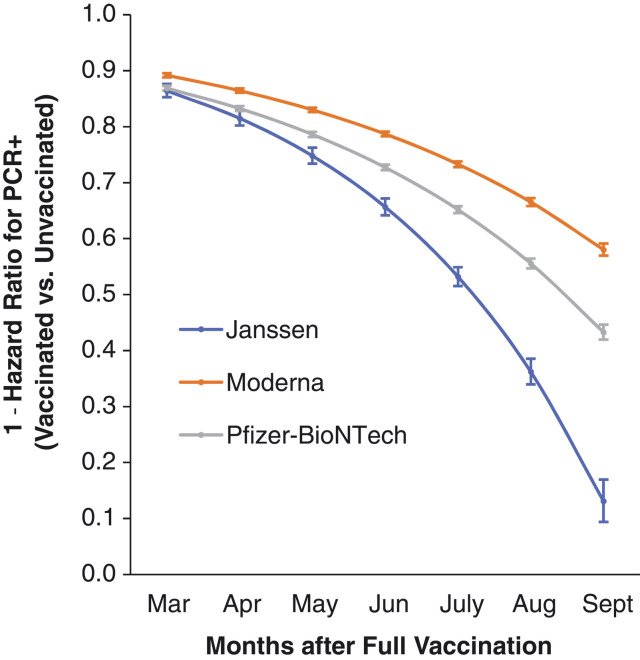
Time-dependent vaccine effectiveness against SARS-CoV-2 infection as estimated from Cox proportional hazards models, adjusted for age, race, ethnicity, sex, and comorbidity score. Vaccine effectiveness presented as (1 – hazard ratio × 100) and 95% CIs. Effectiveness for each month was estimated from contrasts by using product terms for vaccination status by time to most recent RT-PCR assay.

For the period 1 February 2021 to 1 October 2021, vaccine effectiveness against infection (VE-I) declined over time (*P* < 0.01 for time dependence) (Table 1), even after adjusting for age, sex, and comorbidity. VE-I declined for all vaccine types (Fig. 1), with the largest declines for Janssen followed by Pfizer-BioNTech and Moderna. Specifically, in March, VE-I was 86.4% [95% confidence interval (CI), 85.2 to 87.6%) for Janssen, 89.2% (95% CI, 88.8 to 89.6%) for Moderna, and 86.9% (95% CI, 86.5 to 87.3%) for Pfizer-BioNTech. By September, VE-I had declined to 13.1% (95% CI, 9.2 to 16.8%) for Janssen, 58.0% (95% CI, 56.9 to 59.1%) for Moderna, and 43.3% (95% CI, 41.9 to 44.6%) for Pfizer-BioNTech.

**Table 1.** Vaccine effectiveness against SARS-CoV-2 infection by month after vaccination; estimated from Cox proportional hazards models; and adjusted for age, race, ethnicity, sex, and comorbidity score. Adjusted hazard ratio <1.0 indicates lower risk of infection for vaccine, shown compared with unvaccinated. For vaccinated veterans, infection was assessed 15 days after the last vaccine that established full vaccination status. For unvaccinated veterans, infection was assessed beginning in 1 February 2021, coincident with broadscale vaccine eligibility in the VA. Time dependence was tested in Cox proportional hazards models by including product terms for vaccination status (Janssen, Moderna, Pfizer-BioNTech, or unvaccinated) by log(time)—Janssen*log(time), Moderna*log(time), Pfizer-BioNTech*log(time)—and adjusted for age, sex, race, ethnicity, and comorbidity (Charlson Comorbidity score, overweight, type II diabetes, chronic obstructive pulmonary disease, bronchitis, acute respiratory failure, and chronic lung disease). Significance levels for all product terms were *P* < 0.0001. Vaccination status is modeled as time-varying, assigning follow-up time for veterans before the date of full vaccination as “unvaccinated time” and time after the date of full vaccination as “vaccinated time”; vaccination is defined as (i) a single Janssen vaccine, (ii) two Moderna vaccines, or (iii) two Pfizer-BioNTech vaccines.

	**Adjusted** **hazard ratio**	**95% confidence** **interval**		***P* value**
Janssen versus unvaccinated*				
March	0.14	0.12	0.15	<0.01
April	0.19	0.17	0.20	<0.01
May	0.25	0.24	0.27	<0.01
June	0.34	0.33	0.36	<0.01
July	0.47	0.45	0.49	<0.01
August	0.64	0.62	0.66	<0.01
September	0.87	0.83	0.91	<0.01
Moderna versus unvaccinated*				
March	0.11	0.10	0.11	<0.01
April	0.14	0.13	0.14	<0.01
May	0.17	0.17	0.17	<0.01
June	0.21	0.21	0.22	<0.01
July	0.27	0.26	0.27	<0.01
August	0.33	0.33	0.34	<0.01
September	0.42	0.41	0.43	<0.01
Pfizer-BioNTech versus unvaccinated*				
March	0.13	0.13	0.14	<0.01
April	0.17	0.16	0.17	<0.01
May	0.21	0.21	0.22	<0.01
June	0.27	0.27	0.28	<0.01
July	0.35	0.34	0.35	<0.01
August	0.44	0.44	0.45	<0.01
September	0.57	0.55	0.58	<0.01

*Associations at each month were estimated from contrasts by using product terms for vaccination status by time in Cox proportional hazards models, including indicator terms for vaccination status (Janssen, Moderna, or Pfizer-BioNTech) product terms and age, sex, race, ethnicity, and comorbidity (Charlson Comorbidity score, overweight, type II diabetes, chronic obstructive pulmonary disease, bronchitis, acute respiratory failure, and chronic lung disease).

**Fig. 2.**
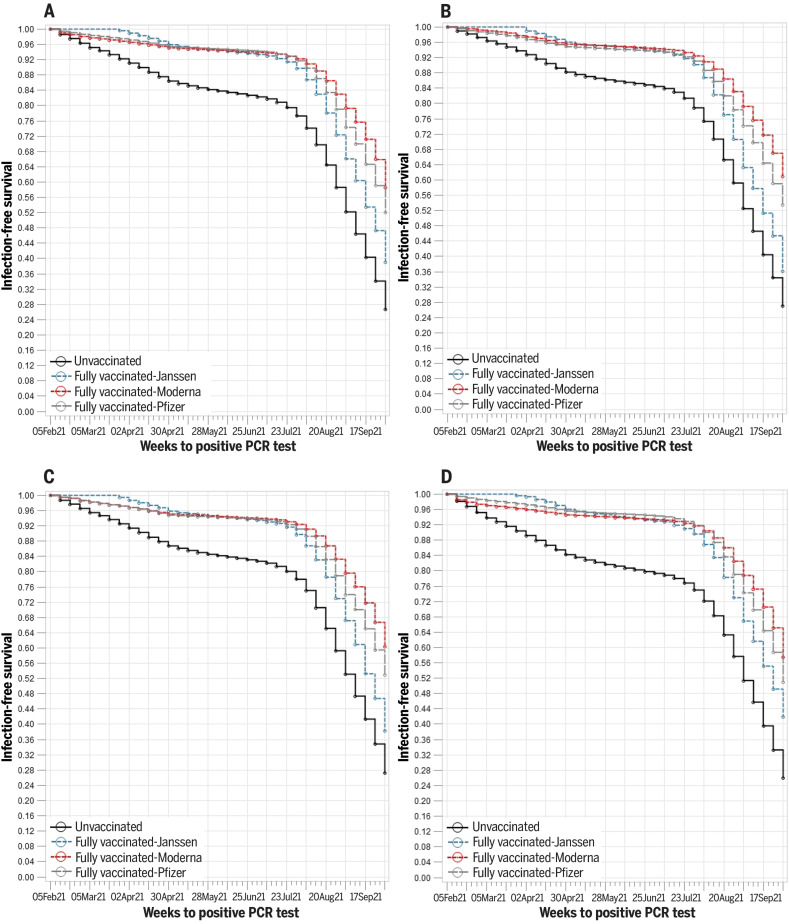
Kaplan-Meier curves illustrating cumulative risk of SARS-CoV-2 infection, by vaccination status and age. (**A**) All ages. (**B**) Age <50 years. (**C**) Age 50 to 64 years. (**D**) Age ≥65 years. The survival function estimates time to infection detected by most recent RT-PCR assay.

As shown in Fig. 2, risk of infection accelerated in both unvaccinated and fully vaccinated veterans beginning in July 2021 and through September 2021, which is consistent with the time dependence observed in the Cox proportional hazards models. This pattern was similar across age groups, and risk of infection was highest for unvaccinated veterans. Veterans who were fully vaccinated with the Moderna vaccine had the lowest risk of infection, followed closely by those who received the Pfizer-BioNTech vaccine, then those who received the Janssen vaccine.

**Fig. 3.**
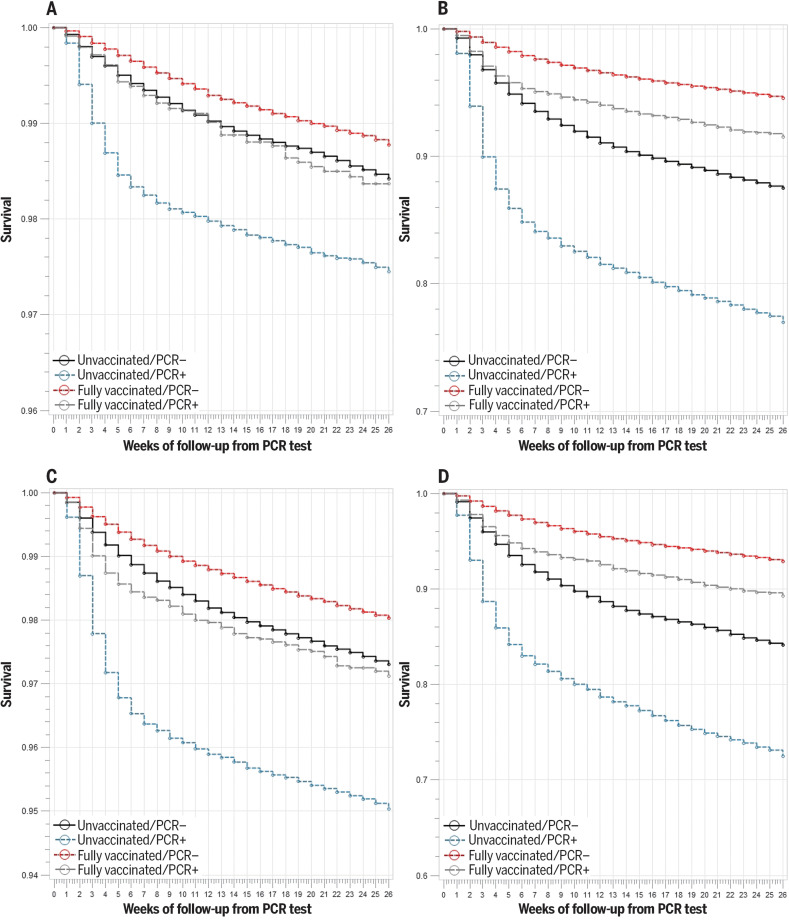
Kaplan-Meier curves illustrating cumulative risk of death due to any cause, by vaccination status and RT-PCR assay. (**A**) Age <65 years. (**B**) Age ≥65 years. (**C**) Charlson Comorbidity Index score <3. (**D**) Charlson Comorbidity Index score ≥3.

Risk of death after SARS-CoV-2 infection was highest in unvaccinated veterans regardless of age and comorbidity (Fig. 3). However, breakthrough infections were not benign, as shown by the higher risk of death in fully vaccinated veterans who became infected compared with vaccinated veterans who remained infection-free.

We observed similar results when examining the time period corresponding to the dominance of the Delta variant (fig. S1). Specifically, among those with a positive PCR test on or after 1 July 2021, vaccination was protective against death, although with some differences by age and vaccine type. For age <65 years, vaccine effectiveness against death (VE-D) was 81.7% (95% CI, 75.7 to 86.2%) for any vaccine, 73.0% (95% CI, 52.0 to 84.8%) for Janssen, 81.5% (95% CI, 70.7 to 88.4%) for Moderna, and 84.3% (95% CI, 76.3 to 89.7%) for Pfizer-BioNTech. For age ≥65 years, VE-D was 71.6% (95% CI, 68.6 to 74.2%) for any vaccine, 52.2% (95% CI, 37.2 to 63.6%) for Janssen, 75.5% (95% CI, 71.8 to 78.7%) for Moderna, and 70.1% (95% CI, 66.1 to 73.6%) for Pfizer-BioNTech.

Benefits of vaccination in reducing risk of SARS-CoV-2 infection and death are clearly supported by this study of more than 780,225 US veterans. However, VE-I declined as risk increased in both unvaccinated and vaccinated veterans, coincident with the emergence and dominance of the Delta variant in the United States. Our analysis by vaccine type—including the Pfizer-BioNTech, Moderna, and Janssen vaccines—suggests a declining VE-I over time, particularly for the Janssen vaccine. Yet, despite increasing risk of infection because of the Delta variant, VE-D remained high, and compared with unvaccinated veterans, those fully vaccinated had a much lower risk of death after infection. These results demonstrate an urgent need to reinstate multiple layers of protection, such as masking and physical distancing—even among vaccinated persons—while also bolstering current efforts to increase vaccination.

Patterns of breakthrough SARS-CoV-2 infection among vaccinated veterans show a worrisome temporal trend, overlapping with the emergence of Delta as the dominant variant in the United States in July 2021. Although others have demonstrated high VE-I and VE-D in veterans during vaccine rollout through mid-March 2021 (*27*), our results suggest that vaccines are less effective in preventing infection associated with the Delta variant. The Delta variant is more infectious than other variants, likely because of increased viral load and transmission before symptoms (*28*). Other US studies (*29*–*31*), many conducted in large health care systems, similarly show declining VE-I as the Delta variant rose to dominance, with notable declines in older adults. For example, two studies conducted at Kaiser Permanente Southern California show that VE-I decreased from 95% at 14 to 60 days to 79% at 151 to 180 days after vaccination for ages 18 to 64 years (*29*), and from 80% at 1 month to 43% at 5 months after vaccination for ages ≥65 years (*31*). Declines in protection against infection with Delta have been observed in Israel (*16*), the UK (*20*, *21*), and Qatar (*32*, *33*).

Endurance of VE-I in the face of the Delta variant in this large, population-based sample was dependent on vaccine type, and this was consistent across all age groups and time since vaccination. Most studies of VE-I have examined Moderna or Pfizer-BioNTech vaccines (*16*, *20*, *21*, *29*–*33*), and our study adds to this literature by showing dramatic declines in VE-I for the Janssen vaccine. Similarly, we found that VE-D for the Janssen vaccine was much lower—about 50%—compared with that of the randomized trial. These findings are consistent with the better neutralizing antibody response observed after vaccination with Moderna or Pfizer-BioNtech compared with Janssen vaccines, and in response to the Delta variant (*34*). In addition, differences in immune response to mRNA vaccines by type of immunity support the more enduring protection against death (through cellular immunity) compared with protection against infection (which is more dependent on antibodies) (*35*).

Our findings on increased risk of death after breakthrough infection provide further support for continuing efforts to discover and implement effective interventions to prevent infection in all persons, including those who have been fully vaccinated. Fully vaccinated veterans were more likely to survive when infected with SARS-CoV-2 (breakthrough infections) compared with unvaccinated veterans who were also infected; this was true even for older age groups, those with more chronic conditions, and during and after the Delta surge in July 2021. However, breakthrough infections still carried some risk, as evidenced by the higher risk of death in vaccinated veterans who were subsequently infected compared with those who were vaccinated but remained infection-free. Breakthrough infections are also a concern for transmission, and the Delta variant in particular results in high viral loads in the nose similar to that of infections in unvaccinated persons (*36*). Because viral load is a key determinant of transmissibility (*37*), the benefit of vaccination is less for the Delta variant compared with the earlier Alpha variant (*38*), suggesting that additional, alternative prevention practices will be essential to reduce infection. Higher risk of death after breakthrough infection implies higher rates of hospitalizations, and these prevention practices will likely also conserve medical resources.

Infection prevention in all persons will have the added, worldwide benefit of reducing the potential for deleterious evolution of the viral genome as the infection is transmitted from person to person (*37*, *39*). However, rates of vaccination—among other viral, social, political, and behavioral parameters—will determine the future evolution of the virus (*37*). Viral evolution may result in more lethal or infectious variants, or variants that escape protection the vaccine, and should be constricted by reducing infection rates.

It is not yet known whether breakthrough infections increase risk of “long COVID” [otherwise known as post-acute sequelae of COVID-19 (PASC)], a constellation of debilitating and lingering symptoms after infection. These symptoms can lead to physiologic disruption of multiple organ systems; substantial disruption of daily life, employment, and mental health; and a higher burden on the health care system (*40*, *41*). Long COVID has been observed as a consequence of both mild and severe infection (*42*), raising the possibility that survivors of breakthrough infections may also be at risk for long COVID. Therefore, prevention of breakthrough infections may avoid the overwhelming, long-term consequences of long COVID due to widespread infection.

As of this report, the scientific community continues to debate booster vaccines in the United States. The FDA authorized Pfizer-BioNTech boosters in September 2021 and Moderna and Janssen boosters in October 2021, and the CDC has made similar recommendations. Although our study does not directly address the benefits and risks of booster vaccines, findings may be interpreted in the context of this ongoing debate. First, VE-I declined most precipitously for the Janssen vaccine, and a booster with one of the mRNA vaccines may result in more durable protection for those initially vaccinated with Janssen. This is further supported by the available, albeit limited, evidence that suggests a stronger antibody response when Janssen vaccination is followed by an mRNA booster (*43*). Second, although their risk of death is much lower, younger veterans (age <65 years) experienced the greatest relative reduction in risk of death associated with vaccination, which suggests that this age group in addition to older adults may benefit from a booster. Early results of the first randomized trial on boosters demonstrates that a booster of Pfizer-BioNTech is 95.6% effective against infection compared with two shots and a placebo (*43*). Some unknowns remain—namely, how effective booster vaccines are against Delta and other emerging variants and how long immunity from a booster may last.

A strength of our study is the use of large-scale, national US Department of Veterans Affairs (VA) data, covering 2.7% of the US population and collected in real time. After transitioning to focus on breakthrough hospitalizations and deaths, the CDC now reports COVID-19 cases, associated hospitalizations, and deaths by vaccination status and age group (available at https://covid.cdc.gov/covid-data-tracker) as weekly rates per 100,000 persons; these data are derived from a network of acute-care hospitals in 14 states and 16 health departments that links case surveillance to immunization systems. Although informative, data lag behind by about 2 months and do not illustrate risk of hospitalization or death after a breakthrough infection. The VA Corporate Data Warehouse was essential to our timely analysis of breakthrough infections and deaths up until 1 October 2021, and moving forward, these data may be used as a tool to comprehensively monitor vaccine effectiveness because other variants are likely to emerge.

Our results should be interpreted in the context of limitations. There are many approaches to evaluating vaccine effectiveness (such as test-negative, case-control, and cohort registry). We required a recent RT-PCR assay to be included in the analysis, a feature of test-negative designs that may minimize confounding owing to health-seeking behavior. However, there may still be differences in testing intervals and frequency by vaccination status. The specific setting or reason for testing is not known, and it is also possible that persons with asymptomatic infections may not have been tested and therefore not included in the analysis. Our sample has proportionately fewer women, although a large number are still included. We did not have information on genotyping of infections to determine the proportion caused by the Delta variant. Patterns of survival for those with a negative PCR test by vaccination status suggests that there are underlying differences in unvaccinated compared with vaccinated persons, and that we did not measure or account for these in our analysis; these differences may contribute to the different risks of death we observed. For example, recent polls suggest that unvaccinated Americans are less willing to adopt COVID-19 precautions, such as mask-wearing and social distancing (*44*). Last, we did not examine VE against hospitalization but used death as a surrogate for clinically severe infection. Our finding that VE-D remained high during the Delta surge is consistent with US studies that show sustained protection against hospitalization (*15*, *30*, *45*).

Although vaccination remains protective against SARS-CoV-2 infection, protection waned as the Delta variant emerged in the United States, and this decline did not differ by age. The Janssen vaccine showed the greatest decline in VE-I. Breakthrough infections were not benign; vaccinated persons who were subsequently infected had a higher risk of death compared with that of vaccinated persons who remained infection-free. Vaccination still provided protection against death in infected persons, and this benefit was observed for the Moderna, Pfizer-BioNTech, and Janssen vaccines during the Delta surge, although the benefit was greater for Moderna and Pfizer-BioNTech compared with Janssen vaccines. Our findings support the conclusion that COVID-19 vaccines remain the most important tool to prevent infection and death. Vaccines should be accompanied by additional measures for both vaccinated and unvaccinated persons, including masking, hand washing, and physical distancing. It is imperative to implement public health interventions, such as strategic testing for control of outbreaks, vaccine passports, employment-based vaccine mandates, vaccination campaigns for eligible children as well as adults, and consistent messaging from public health leadership in the face of increased risk of infection from the Delta and other emerging variants.

## Supplementary Material

20211104-1

## Supplementary Materials

### Other Supplementary Material for this manuscript includes the following:

MDAR Reproducibility Checklist
