# Supplementary material for: SARS-CoV-2 vaccine protection and deaths among US veterans during 2021
Source: Science. 2021 Nov 4;375(6578):331–6. doi: 10.1126/science.abm0620 (PMC9836205; doi:10.1126/science.abm0620)
Supplement: 20211104-1 [file science.abm0620.v1.pdf]

Cite as: B. A. Cohn *et al.*, *Science* 10.1126/science.abm0620 (2021).

SARS-CoV-2 vaccine protection and deaths among US veterans during 2021

Barbara A. Cohn[1]†, Piera M. Cirillo[1,2]†, Caitlin C. Murphy[3]†, Nickilou Y. Krigbaum[1,2], Arthur W. Wallace[2,4]*

[1]Public Health Institute; Oakland, CA, USA. [2]Veterans Affairs Medical Center in San Francisco; San Francisco, CA, USA. [3]School of Public Health, University of Texas Health Science Center at Houston; Houston, TX, USA. [4]University of California, San Francisco; San Francisco, CA, USA.

†These authors contributed equally to this work.

***Corresponding author. Email: art.wallace@ va.gov**

We report SARS-CoV-2 vaccine effectiveness against infection (VE-I) and death (VE-D) by vaccine type (*n* = 780,225) in the Veterans Health Administration, covering 2.7% of the U.S. population. From February to October 2021, VE-I declined from 87.9% to 48.1%, and the decline was greatest for the Janssen vaccine resulting in a VE-I of 13.1%. Although breakthrough infection increased risk of death, vaccination remained protective against death in persons who became infected during the Delta surge. From July to October 2021, VE-D for age <65 years was 73.0% for Janssen, 81.5% for Moderna, and 84.3% for Pfizer-BioNTech; VE-D for age ≥65 years was 52.2% for Janssen, 75.5% for Moderna, and 70.1% for Pfizer-BioNTech. Findings support continued efforts to increase vaccination, booster campaigns, and multiple, additional layers of protection against infection.

The messenger RNA vaccines BNT162b2 (Pfizer–BioNTech) and mRNA-1273 (Moderna) and the viral vector vaccine JNJ-78436735 (Janssen) have effectively prevented clinically significant disease caused by severe acute respiratory syndrome coronavirus 2 (SARS-CoV-2) since their rollout in the U.S. in late 2020 (*1*, *2*). Vaccines have also reduced the incidence of asymptomatic infection and associated infectivity (*3*). However, by July 2021, the U.S. experienced a surge in cases of COVID-19, dominated by the B.1.617.2 (Delta) variant (*4*, *5*). Initial reports, including follow-up of the Pfizer-BioNTech and Moderna trials (*6*–*8*), suggested sustained vaccine protection (*9*), but three reports of the U.S. Centers for Disease Control (CDC) in August 2021 (*10*–*12*) demonstrated protection against infection had declined in mid-summer as the Delta variant rose to dominance; protection against hospitalization and death remained high (*13*–*15*). Breakthrough infections, illness, hospitalizations, and deaths have since continued to emerge in vaccine recipients.

This phenomenon has been most comprehensively monitored in Israel, where high levels of transmission of the Delta variant led to a resurgent outbreak in mid-June 2021 (*16*), despite a successful nationwide campaign to vaccinate the population (*17*). Israel authorized boosters of the Pfizer-BioNTech vaccine for adults age ≥ 60 years in July 2021 and extended this authorization to adults age ≥ 50 years in August 2021 (*18*). Rates of infection and severe illness subsequently declined in those who received a booster (*19*). Based largely on these data, as well as data from the UK (*20*, *21*), the U.S. Food and Drug Administration (FDA) authorized boosters of the Pfizer-BioNTech vaccine for older (age ≥ 65 years) and higher-risk adults in September 2021 (*22*); they similarly authorized boosters of the Moderna vaccine in October 2021, as well as boosters for all recipients of the Janssen vaccine (*23*).

The debate over boosters in the U.S. (*24*) has laid bare the limitations of its public health infrastructure: national data on vaccine breakthrough are inadequate. The CDC transitioned in May 2021 from monitoring all breakthrough infections to focus on identifying and investigating only hospitalized or fatal cases due to any cause, including causes not related to COVID-19 (*25*). Some data on vaccinations, infections, and deaths are collected through a patchwork of local health departments (*10*), but these data are frequently out of date and difficult to aggregate at the national level. Here, we address this gap and examine SARS-CoV-2 infection and deaths by vaccination status in 780,225 Veterans during the period February 1, 2021 to October 1, 2021, encompassing the emergence and dominance of the Delta variant in the U.S.

Table S1 shows the distribution of SARS-CoV-2 infection by demographics, comorbidity, and vaccination status for February 1, 2021 to October 1, 2021 (*n* = 780,225). The percentage of PCR test positivity is higher in Veterans who were unvaccinated (25.8%), female (18.2%), Hispanic (18.0%), American Indian/Alaska Native (18.2%) or Native Hawaiian/Pacific Islander (17.8%), age <50 years at RT-PCR assay (22.2%), and had a lower comorbidity score (19.2% for Charlson Comorbidity Index = 0) (*26*). 58,967 positive PCR tests

occurred in 498,148 fully vaccinated Veterans. Table S2 shows the distribution of vaccine type by demographics. Vaccine type differed by age: younger (age < 50 years) Veterans were more likely to have received the Janssen vaccine than either Moderna or Pfizer- BioNTech.

For the period February 1, 2021 to October 1, 2021, vaccine effectiveness against infection (VE-I) declined over time ($P < 0.01$ for time dependence, Table 1), even after adjusting for age, sex, and comorbidity. VE-I declined for all vaccine types (Fig. 1), with the largest declines for Janssen followed by Pfizer-BioNTech and Moderna. Specifically, in March, VE-I was 86.4% (95% CI: 85.2% to 87.6%) for Janssen; 89.2% (95% CI: 88.8% to 89.6%) for Moderna; and 86.9% (95% CI: 86.5% to 87.3%) for Pfizer-BioNTech. By September, VE-I had declined to 13.1% (95% CI: 9.2% to 16.8%) for Janssen; 58.0% (95% CI: 56.9% to 59.1%) for Moderna; and 43.3% (95% CI: 41.9% to 44.6%) for Pfizer-BioNTech.

As shown in Fig. 2, risk of infection accelerated in both unvaccinated and fully vaccinated Veterans beginning in July 2021 and through September 2021, consistent with the time dependence observed in the Cox proportional hazards models. This pattern was similar across age groups, and risk of infection was highest for unvaccinated Veterans. Veterans who were fully vaccinated with the Moderna vaccine had the lowest risk of infection, followed closely by those who received the Pfizer-BioNTech vaccine, then those who received the Janssen vaccine.

Risk of death after SARS-CoV-2 infection was highest in unvaccinated Veterans regardless of age and comorbidity (Fig. 3). However, breakthrough infections were not benign, as shown by the higher risk of death in fully vaccinated Veterans who became infected compared to vaccinated Veterans who remained infection-free.

We observed similar results when examining the time period corresponding to the dominance of the Delta variant (fig. S1). Specifically, among those with a positive PCR test on or after July 1, 2021, vaccination was protective against death, although with some differences by age and vaccine type. For age <65 years, vaccine effectiveness against death (VE-D) was 81.7% (95% CI: 75.7% to 86.2%) for any vaccine; 73.0% (95% CI: 52.0% to 84.8%) for Janssen; 81.5% (95% CI: 70.7% to 88.4%) for Moderna; and 84.3% (95% CI: 76.3% to 89.7%) for Pfizer-BioNTech. For age ≥65 years, VE-D was 71.6% (95% CI: 68.6% to 74.2%) for any vaccine; 52.2% (95% CI: 37.2% to 63.6%) for Janssen; 75.5% (95% CI: 71.8% to 78.7%) for Moderna; and 70.1% (95% CI: 66.1% to 73.6%) for Pfizer-BioNTech.

Benefits of vaccination in reducing risk of SARS-CoV-2 infection and death are clearly supported by this study of more than 780,225 U.S. Veterans. However, VE-I declined as risk increased in both unvaccinated and vaccinated Veterans, coincident with the emergence and dominance of the Delta variant in the U.S. Our analysis by vaccine type, including the Pfizer-BioNTech, Moderna, and Janssen vaccines, suggests declining VE-I over time, particularly for the Janssen vaccine. Yet, despite increasing risk of infection due to the Delta variant, VE-D remained high, and compared to unvaccinated Veterans, those fully vaccinated had a much lower risk of death after infection. These results demonstrate an urgent need to reinstate multiple layers of protection, such as masking and physical distancing – even among vaccinated persons – while also bolstering current efforts to increase vaccination.

Patterns of breakthrough SARS-CoV-2 infection among vaccinated Veterans show a worrisome temporal trend, overlapping with the emergence of Delta as the dominant variant in the U.S. in July 2021. Although others have demonstrated high VE-I and VE-D in Veterans during vaccine rollout through mid-March 2021 (*27*), our results suggest vaccines are less effective in preventing infection associated with the Delta variant. The Delta variant is more infectious than other variants, likely due to increased viral load and transmission prior to symptoms (*28*). Other U.S. studies (*29*–*31*), many conducted in large healthcare systems, similarly show declining VE-I as the Delta variant rose to dominance, with notable declines in older adults. For example, two studies conducted in Kaiser Permanente Southern California show VE-I decreased from 95% at 14-60 days to 79% at 151-180 days after vaccination for ages 18-64 years (*29*), and from 80% at 1 month to 43% at 5 months after vaccination for ages ≥65 years (*31*). Declines in protection against infection with Delta have been observed in Israel (*16*), the UK (*20*, *21*), and Qatar (*32*, *33*).

Importantly, endurance of VE-I in the face of the Delta variant in this large, population-based sample was dependent upon vaccine type, and this was consistent across all age groups and time since vaccination. Most studies of VE-I have examined Moderna or Pfizer-BioNTech vaccines (*16*, *20*, *21*, *29*–*33*), and our study adds to this literature by showing dramatic declines in VE-I for the Janssen vaccine. Similarly, we found VE-D for the Janssen vaccine was much lower– about 50% – compared to the randomized trial. These findings are consistent with the better neutralizing antibody response observed following vaccination with Moderna or Pfizer-BioNtech compared to Janssen vaccines, and in response to the Delta variant (*34*). In addition, differences in immune response to mRNA vaccines by type of immunity support the more enduring protection against death (via cellular immunity) compared to protection against infection (more dependent on antibodies) (*35*).

Our findings on increased risk of death following breakthrough infection provide further support for continuing efforts to discover and implement effective interventions to prevent infection in all persons, including those who have been fully vaccinated. Fully vaccinated Veterans were more likely to survive when infected with SARS-CoV-2 (i.e.,

breakthrough infections) compared to unvaccinated Veterans who were also infected – this was true even for older age groups, those with more chronic conditions, and during and after the Delta surge in July 2021. However, breakthrough infections still carried some risk, as evidenced by the higher risk of death in vaccinated Veterans who were subsequently infected compared to those who were vaccinated but remained infection-free. Breakthrough infections are also a concern for transmission, and in particular with the Delta variant is it results in high viral loads in the nose similar to infections in unvaccinated persons (*36*). Because viral load is a key determinant of transmissibility (*37*), the benefit of vaccination is less for the Delta variant compared to the earlier Alpha variant (*38*), suggesting that additional, alternative prevention practices will be essential to reduce infection. Higher risk of death following breakthrough infection implies higher rates of hospitalizations, and these prevention practices will likely also conserve medical resources.

Infection prevention in all persons will have the added, world-wide benefit of reducing the potential for deleterious evolution of the viral genome as the infection is transmitted from person to person (*37*, *39*). However, rates of vaccination, among other viral, social, political and behavioral parameters, will determine the future evolution of the virus (*37*). Viral evolution may result in more lethal or infectious variants, or variants that escape protection the vaccine, and should be constricted by reducing infection rates.

It is not yet known whether breakthrough infections increase risk of long COVID (otherwise known as post-acute sequelae of COVID-19 or PASC), a constellation of debilitating and lingering symptoms following infection. These symptoms can lead to physiologic disruption of multiple organ systems, substantial disruption of daily life, employment, and mental health, and a higher burden on the healthcare system (*40*, *41*). Long COVID has been observed as a consequence of both mild and severe infection (*42*), raising the possibility that survivors of breakthrough infections may also be at risk for long COVID. Therefore, prevention of breakthrough infections may avoid the overwhelming, long-term consequences of long COVID due to widespread infection.

As of this report, the scientific community continues to debate booster vaccines in the U.S. The FDA authorized Pfizer-BioNTech boosters in September 2021 and Moderna and Janssen boosters in October 2021, and the CDC has made similar recommendations. Although our study does not directly address the benefits and risks of booster vaccines, findings may be interpreted in the context of this ongoing debate. First, VE-I declined most precipitously for the Janssen vaccine, and a booster with one of the mRNA vaccines may result in more durable protection for those initially vaccinated with Janssen. This is further supported by the available, albeit limited, evidence suggesting a stronger antibody response when Janssen vaccination is followed by an mRNA booster (*43*). Second, although their risk of death is much lower, younger Veterans (age <65 years) experienced the greatest relative reduction in risk of death associated with vaccination, suggesting this age group may benefit from a booster in addition to older adults. Early results of the first randomized trial on boosters demonstrates a booster of Pfizer-BioNTech is 95.6% effective against infection compared to two shots and a placebo (*43*). Some unknowns remain, namely how effective booster vaccines are against Delta and other emerging variants and how long immunity from a booster may last.

An important strength of our study is the use of large-scale, national VA data, covering 2.7% of the U.S. population and collected in real time. After transitioning to focus on breakthrough hospitalizations and deaths, the CDC now reports COVID-19 cases, associated hospitalizations, and deaths by vaccination status and age group (available at: https://covid.cdc.gov/covid-data-tracker) as weekly rates per 100,000 persons; these data are derived from a network of acute-care hospitals in 14 states and 16 health departments linking case surveillance to immunization systems. Although informative, data lag behind by about two months, and they do not illustrate risk of hospitalization or death *after* a breakthrough infection. The VA CDW was essential to our timely analysis of breakthrough infections and deaths up until October 1, 2021, and moving forward, these data may be used as tool to comprehensively monitor vaccine effectiveness as other variants are likely to emerge.

Our results should be interpreted in the context of limitations. There are many approaches to evaluating vaccine effectiveness (e.g., test-negative, case-control, cohort registry). We required a recent RT-PCR assay to be included in the analysis, a feature of test-negative designs that may minimize confounding due to health seeking behavior. However, there may still be differences in testing intervals and frequency by vaccination status. The specific setting or reason for testing is not known, and it is also possible that persons with asymptomatic infections may not have been tested and therefore not included in the analysis. Our sample has proportionately fewer women, although a large number are still included. We did not have information on genotyping of infections to determine the proportion caused by the Delta variant. Patterns of survival for those with a negative PCR test by vaccination status suggests there are underlying differences in unvaccinated compared to vaccinated persons, and that we did not measure or account for in our analysis; these differences may contribute to the different risks of death we observed. For example, recent polls suggest unvaccinated Americans are less willing to adopt COVID-19 precautions, such as masking wearing and social distancing (*44*). Finally, we did not examine VE against hospitalization but used death as a surrogate for clinical significance of infection. Our finding that VE-D

remained high during the Delta surge is consistent with U.S. studies showing sustained protection against hospitalization (*15*, *30*, *45*).

In summary, although vaccination remains protective against SARS-CoV-2 infection, protection waned as the Delta variant emerged in the U.S., and this decline did not differ by age. The Janssen vaccine showed the greatest decline in VE-I. Breakthrough infections were not benign as vaccinated persons and who were subsequently infected had a higher risk of death compared to vaccinated persons who remained infection-free. Importantly, vaccination still provided protection against death in infected persons, and this benefit was observed for the Moderna, Pfizer-BioNTech, and Janssen vaccines during the Delta surge, although the benefit was greater for Moderna and Pfizer-BioNTech compared to Janssen vaccines. Our findings support the conclusion that COVID-19 vaccines remain the most important tool to prevent infection and death. Vaccines should be accompanied by additional measures for both vaccinated and unvaccinated persons, including masking, hand washing, and physical distancing. It is essential to implement public health interventions, such as strategic testing for control of outbreaks, vaccine passports, employment-based vaccine mandates, vaccination campaigns for eligible children as well as adults, and consistent messaging from public health leadership in the face of increased risk of infection due to the Delta and other emerging variants.

REFERENCES AND NOTES

1. T. Pilishvili, K. E. Fleming-Dutra, J. L. Farrar, R. Gierke, N. M. Mohr, D. A. Talan, A. Krishnadasan, K. K. Harland, H. A. Smithline, P. C. Hou, L. C. Lee, S. C. Lim, G. J. Moran, E. Krebs, M. Steele, D. G. Beiser, B. Faine, J. P. Haran, U. Nandi, W. A. Schrading, B. Chinnock, D. J. Henning, F. LoVecchio, J. Nadle, D. Barter, M. Brackney, A. Britton, K. Marceaux-Galli, S. Lim, E. C. Phipps, G. Dumyati, R. Pierce, T. M. Markus, D. J. Anderson, A. K. Debes, M. Lin, J. Mayer, H. M. Babcock, N. Safdar, M. Fischer, R. Singleton, N. Chea, S. S. Magill, J. Verani, S. Schrag, A. Yousaf, Y. Chung, J. Onukwube, W. Xing, B. Clinansmith, L. Uribe, K. E. Poronsky, D. M. Hashimoto, M. Bahamon, M. St. Romain, E. Kean, A. Stubbs, S. Roy, G. Volturo, J. Galbraith, J. C. Crosby, M. R. Fuentes, M. Mulrow, J. Lee, H. Johnston, A. J. Hansen, S. K. Fridkin, L. E. Wilson, S. Lovett, M. Christian, C. Myers, V. L. S. Ocampo, K. Talbot, J. Seidelman, A. M. Milstone, M. Hayden, M. Samore, J. H. Kwon, D. Shirley, D. Dillard, J. Dobson; Vaccine Effectiveness Among Healthcare Personnel Study Team, Interim Estimates of Vaccine Effectiveness of Pfizer-BioNTech and Moderna COVID-19 Vaccines Among Health Care Personnel - 33 U.S. Sites, January-March 2021. *MMWR Morb. Mortal. Wkly. Rep.* **70**, 753–758 (2021). doi:10.15585/mmwr.mm7020e2 Medline
2. A. Christie, S. J. Henley, L. Mattocks, R. Fernando, A. Lansky, F. B. Ahmad, J. Adjemian, R. N. Anderson, A. M. Binder, K. Carey, D. L. Dee, T. Dias, W. M. Duck, D. M. Gaughan, B. C. Lyons, A. D. McNaghten, M. M. Park, H. Reses, L. Rodgers, K. Van Santen, D. Walker, M. J. Beach, Decreases in COVID-19 Cases, Emergency Department Visits, Hospital Admissions, and Deaths Among Older Adults Following the Introduction of COVID-19 Vaccine - United States, September 6, 2020-May 1, 2021. *MMWR Morb. Mortal. Wkly. Rep.* **70**, 858–864 (2021). doi:10.15585/mmwr.mm7023e2 Medline
3. M. G. Thompson, J. L. Burgess, A. L. Naleway, H. L. Tyner, S. K. Yoon, J. Meece, L. E. W. Olsho, A. J. Caban-Martinez, A. Fowlkes, K. Lutrick, J. L. Kuntz, K. Dunnigan, M. J. Odean, K. T. Hegmann, E. Stefanski, L. J. Edwards, N. Schaefer-Solle, L. Grant, K. Ellingson, H. C. Groom, T. Zunie, M. S. Thiese, L. Ivacic, M. G. Wesley, J. M. Lamberte, X. Sun, M. E. Smith, A. L. Phillips, K. D. Groover, Y. M. Yoo, J. Gerald, R. T. Brown, M. K. Herring, G. Joseph, S. Beitel, T. C. Morrill, J. Mak, P. Rivers, K. M. Harris, D. R. Hunt, M. L. Arvay, P. Kutty, A. M. Fry, M. Gaglani, Interim Estimates of Vaccine Effectiveness of BNT162b2 and mRNA-1273 COVID-19 Vaccines in Preventing SARS-CoV-2 Infection Among Health Care Personnel, First Responders, and Other Essential and Frontline Workers - Eight U.S. Locations, December 2020-March 2021. *MMWR Morb. Mortal. Wkly. Rep.* **70**, 495–500 (2021). doi:10.15585/mmwr.mm7013e3 Medline
4. C. M. Brown, J. Vostok, H. Johnson, M. Burns, R. Gharpure, S. Sami, R. T. Sabo, N. Hall, A. Foreman, P. L. Schubert, G. R. Gallagher, T. Fink, L. C. Madoff, S. B. Gabriel, B. MacInnis, D. J. Park, K. J. Siddle, V. Harik, D. Arvidson, T. Brock-Fisher, M. Dunn, A. Kearns, A. S. Laney, Outbreak of SARS-CoV-2 Infections, Including COVID-19 Vaccine Breakthrough Infections, Associated with Large Public Gatherings - Barnstable County, Massachusetts, July 2021. *MMWR Morb. Mortal. Wkly. Rep.* **70**, 1059–1062 (2021). doi:10.15585/mmwr.mm7031e2 Medline
5. R. Herlihy, W. Bamberg, A. Burakoff, N. Alden, R. Severson, E. Bush, B. Kawasaki, B. Berger, E. Austin, M. Shea, E. Gabrieloff, S. Matzinger, A. Burdorf, J. Nichols, K. Goode, A. Cilwick, C. Stacy, E. Staples, G. Stringer, Rapid Increase in Circulation of the SARS-CoV-2 B.1.617.2 (Delta) Variant - Mesa County, Colorado, April-June 2021. *MMWR Morb. Mortal. Wkly. Rep.* **70**, 1084–1087 (2021). doi:10.15585/mmwr.mm7032e2 Medline
6. S. J. Thomas, E. D. Moreira Jr., N. Kitchin, J. Absalon, A. Gurtman, S. Lockhart, J. L. Perez, G. Pérez Marc, F. P. Polack, C. Zerbini, R. Bailey, K. A. Swanson, X. Xu, S. Roychoudhury, K. Koury, S. Bouguermouh, W. V. Kalina, D. Cooper, R. W. Frenck Jr., L. L. Hammitt, Ö. Türeci, H. Nell, A. Schaefer, S. Ünal, Q. Yang, P. Liberator, D. B. Tresnan, S. Mather, P. R. Dormitzer, U. Şahin, W. C. Gruber, K. U. Jansen; C4591001 Clinical Trial Group, Safety and Efficacy of the BNT162b2 mRNA Covid-19 Vaccine through 6 Months. *N. Engl. J. Med.* 10.1056/NEJMoa2110345 (2021). doi:10.1056/NEJMoa2110345 Medline
7. N. Doria-Rose, M. S. Suthar, M. Makowski, S. O'Connell, A. B. McDermott, B. Flach, J. E. Ledgerwood, J. R. Mascola, B. S. Graham, B. C. Lin, S. O'Dell, S. D. Schmidt, A. T. Widge, V.-V. Edara, E. J. Anderson, L. Lai, K. Floyd, N. G. Rouphael, V. Zarnitsyna, P. C. Roberts, M. Makhene, W. Buchanan, C. J. Luke, J. H. Beigel, L. A. Jackson, K. M. Neuzil, H. Bennett, B. Leav, J. Albert, P. Kunwar; mRNA-1273 Study Group, Antibody Persistence through 6 Months after the Second Dose of mRNA-1273 Vaccine for Covid-19. *N. Engl. J. Med.* **384**, 2259–2261 (2021). doi:10.1056/NEJMc2103916 Medline
8. H. M. El Sahly, L. R. Baden, B. Essink, S. Doblecki-Lewis, J. M. Martin, E. J. Anderson, T. B. Campbell, J. Clark, L. A. Jackson, C. J. Fichtenbaum, M. Zervos, B. Rankin, F. Eder, G. Feldman, C. Kennelly, L. Han-Conrad, M. Levin, K. M. Neuzil, L. Corey, P. Gilbert, H. Janes, D. Follmann, M. Marovich, L. Polakowski, J. R. Mascola, J. E. Ledgerwood, B. S. Graham, A. August, H. Clouting, W. Deng, S. Han, B. Leav, D. Manzo, R. Pajon, F. Schödel, J. E. Tomassini, H. Zhou, J. Miller; COVE Study Group, Efficacy of the mRNA-1273 SARS-CoV-2 Vaccine at Completion of Blinded Phase. *N. Engl. J. Med.* 10.1056/NEJMoa2113017 (2021). doi:10.1056/NEJMoa2113017 Medline
9. J. M. Polinski *et al.*, Effectiveness of the Single-Dose Ad26.COV2.S COVID Vaccine. *medRxiv*, 2021.2009.2010.21263385 (2021). doi:10.1101/2021.09.10.21263385.
10. E. S. Rosenberg, D. R. Holtgrave, V. Dorabawila, M. Conroy, D. Greene, E. Lutterloh, B. Backenson, D. Hoefer, J. Morne, U. Bauer, H. A. Zucker, New COVID-19 Cases and Hospitalizations Among Adults, by Vaccination Status - New York, May 3-July 25, 2021. *MMWR Morb. Mortal. Wkly. Rep.* **70**, 1306–1311 (2021). doi:10.15585/mmwr.mm7037a7 Medline
11. S. Nanduri, T. Pilishvili, G. Derado, M. M. Soe, P. Dollard, H. Wu, Q. Li, S. Bagchi, H. Dubendris, R. Link-Gelles, J. A. Jernigan, D. Budnitz, J. Bell, A. Benin, N. Shang, J. R. Edwards, J. R. Verani, S. J. Schrag, Effectiveness of Pfizer-BioNTech and Moderna Vaccines in Preventing SARS-CoV-2 Infection Among Nursing Home Residents Before and During Widespread Circulation of the SARS-CoV-2 B.1.617.2 (Delta) Variant - National Healthcare Safety Network, March 1-August 1, 2021. *MMWR Morb. Mortal. Wkly. Rep.* **70**, 1163–1166 (2021). doi:10.15585/mmwr.mm7034e3 Medline
12. A. Fowlkes, M. Gaglani, K. Groover, M. S. Thiese, H. Tyner, K. Ellingson; HEROES-RECOVER Cohorts, Effectiveness of COVID-19 Vaccines in Preventing SARS-CoV-2 Infection Among Frontline Workers Before and During B.1.617.2 (Delta) Variant Predominance - Eight U.S. Locations, December 2020-August 2021. *MMWR Morb. Mortal. Wkly. Rep.* **70**, 1167–1169 (2021). doi:10.15585/mmwr.mm7034e4 Medline

13. M. W. Tenforde, W. H. Self, E. A. Naioti, A. A. Ginde, D. J. Douin, S. M. Olson, H. K. Talbot, J. D. Casey, N. M. Mohr, A. Zepeski, M. Gaglani, T. McNeal, S. Ghamande, N. I. Shapiro, K. W. Gibbs, D. C. Files, D. N. Hager, A. Shehu, M. E. Prekker, H. L. Erickson, M. N. Gong, A. Mohamed, D. J. Henning, J. S. Steingrub, I. D. Peltan, S. M. Brown, E. T. Martin, A. S. Monto, A. Khan, C. L. Hough, L. W. Busse, C. C. Ten Lohuis, A. Duggal, J. G. Wilson, A. J. Gordon, N. Qadir, S. Y. Chang, C. Mallow, C. Rivas, H. M. Babcock, J. H. Kwon, M. C. Exline, N. Halasa, J. D. Chappell, A. S. Lauring, C. G. Grijalva, T. W. Rice, I. D. Jones, W. B. Stubblefield, A. Baughman, K. N. Womack, C. J. Lindsell, K. W. Hart, Y. Zhu, M. Stephenson, S. J. Schrag, M. Kobayashi, J. R. Verani, M. M. Patel, N. Calhoun, K. Murthy, J. Herrick, A. McKillop, E. Hoffman, M. Zayed, M. Smith, N. Settele, J. Ettlinger, E. Priest, J. Thomas, A. Arroliga, M. Beeram, R. Kindle, L.-A. Kozikowski, L. De Souza, S. Ouellette, S. Thornton-Thompson, P. Tyler, O. Mehkri, K. Ashok, S. Gole, A. King, B. Poynter, N. Stanley, A. Hendrickson, E. Maruggi, T. Scharber, J. Jorgensen, R. Bowers, J. King, V. Aston, B. Armbruster, R. E. Rothman, R. Nair, J.-T. T. Chen, S. Karow, E. Robart, P. N. Maldonado, M. Khan, P. So, J. Levitt, C. Perez, A. Visweswaran, J. Roque, A. Rivera, T. Frankel, M. Howell, J. Friedel, J. Goff, D. Huynh, M. Tozier, C. Driver, M. Carricato, A. Foster, P. Nassar, L. Stout, Z. Sibenaller, A. Walter, J. Mares, L. Olson, B. Clinansmith, C. Rivas, H. Gershengorn, E. J. McSpadden, R. Truscon, A. Kaniclides, L. Thomas, R. Bielak, W. D. Valvano, R. Fong, W. J. Fitzsimmons, C. Blair, A. L. Valesano, J. Gilbert, C. D. Crider, K. A. Steinbock, T. C. Paulson, L. A. Anderson, C. Kampe, J. Johnson, R. McHenry, M. Blair, D. Conway, M. LaRose, L. Landreth, M. Hicks, L. Parks, J. Bongu, D. McDonald, C. Cass, S. Seiler, D. Park, T. Hink, M. Wallace, C.-A. Burnham, O. G. Arter; IVY Network Investigators; IVY Network, Sustained Effectiveness of Pfizer-BioNTech and Moderna Vaccines Against COVID-19 Associated Hospitalizations Among Adults - United States, March-July 2021. *MMWR Morb. Mortal. Wkly. Rep.* **70**, 1156–1162 (2021). doi:10.15585/mmwr.mm7034e2 Medline
14. H. M. Scobie, A. G. Johnson, A. B. Suthar, R. Severson, N. B. Alden, S. Balter, D. Bertolino, D. Blythe, S. Brady, B. Cadwell, I. Cheng, S. Davidson, J. Delgadillo, K. Devinney, J. Duchin, M. Duwell, R. Fisher, A. Fleischauer, A. Grant, J. Griffin, M. Haddix, J. Hand, M. Hanson, E. Hawkins, R. K. Herlihy, L. Hicks, C. Holtzman, M. Hoskins, J. Hyun, R. Kaur, M. Kay, H. Kidrowski, C. Kim, K. Komatsu, K. Kugeler, M. Lewis, B. C. Lyons, S. Lyons, R. Lynfield, K. McCaffrey, C. McMullen, L. Milroy, S. Meyer, L. Nolen, M. R. Patel, S. Pogosjans, H. E. Reese, A. Saupe, J. Sell, T. Sokol, D. Sosin, E. Stanislawski, K. Stevens, H. Vest, K. White, E. Wilson, A. MacNeil, M. D. Ritchey, B. J. Silk, Monitoring Incidence of COVID-19 Cases, Hospitalizations, and Deaths, by Vaccination Status - 13 U.S. Jurisdictions, April 4-July 17, 2021. *MMWR Morb. Mortal. Wkly. Rep.* **70**, 1284–1290 (2021). doi:10.15585/mmwr.mm7037e1 Medline
15. W. H. Self, M. W. Tenforde, J. P. Rhoads, M. Gaglani, A. A. Ginde, D. J. Douin, S. M. Olson, H. K. Talbot, J. D. Casey, N. M. Mohr, A. Zepeski, T. McNeal, S. Ghamande, K. W. Gibbs, D. C. Files, D. N. Hager, A. Shehu, M. E. Prekker, H. L. Erickson, M. N. Gong, A. Mohamed, D. J. Henning, J. S. Steingrub, I. D. Peltan, S. M. Brown, E. T. Martin, A. S. Monto, A. Khan, C. L. Hough, L. W. Busse, C. C. Ten Lohuis, A. Duggal, J. G. Wilson, A. J. Gordon, N. Qadir, S. Y. Chang, C. Mallow, C. Rivas, H. M. Babcock, J. H. Kwon, M. C. Exline, N. Halasa, J. D. Chappell, A. S. Lauring, C. G. Grijalva, T. W. Rice, I. D. Jones, W. B. Stubblefield, A. Baughman, K. N. Womack, C. J. Lindsell, K. W. Hart, Y. Zhu, L. Mills, S. N. Lester, M. M. Stumpf, E. A. Naioti, M. Kobayashi, J. R. Verani, N. J. Thornburg, M. M. Patel, N. Calhoun, K. Murthy, J. Herrick, A. McKillop, E. Hoffman, M. Zayed, M. Smith, N. Seattle, J. Ettlinger, E. Priest, J. Thomas, A. Arroliga, M. Beeram, R. Kindle, L.-A. Kozikowski, L. De Souza, S. Ouellette, S. Thornton-Thompson, O. Mehkri, K. Ashok, S. Gole, A. King, B. Poynter, N. Stanley, A. Hendrickson, E. Maruggi, T. Scharber, J. Jorgensen, R. Bowers, J. King, V. Aston, B. Armbruster, R. E. Rothman, R. Nair, J.-T. T. Chen, S. Karow, E. Robart, P. N. Maldonado, M. Khan, P. So, J. Levitt, C. Perez, A. Visweswaran, J. Roque, A. Rivera, L. Angeles, T. Frankel, L. Angeles, J. Goff, D. Huynh, M. Howell, J. Friedel, M. Tozier, C. Driver, M. Carricato, A. Foster, P. Nassar, L. Stout, Z. Sibenaller, A. Walter, J. Mares, L. Olson, B. Clinansmith, C. Rivas, H. Gershengorn, E. J. McSpadden, R. Truscon, A. Kaniclides, L. Thomas, R. Bielak, W. D. Valvano, R. Fong, W. J. Fitzsimmons, C. Blair, A. L. Valesano, J. Gilbert, C. D. Crider, K. A. Steinbock, T. C. Paulson, L. A. Anderson, C. Kampe, J. Johnson, R. McHenry, M. Blair, D. Conway, M. LaRose, L. Landreth, M. Hicks, L. Parks, J. Bongu, D. McDonald, C. Cass, S. Seiler, D. Park, T. Hink, M. Wallace, C.-A. Burnham, O. G. Arter; IVY Network, Comparative Effectiveness of Moderna, Pfizer-BioNTech, and Janssen (Johnson & Johnson) Vaccines in Preventing COVID-19 Hospitalizations Among Adults Without Immunocompromising Conditions - United States, March-August 2021. *MMWR Morb. Mortal. Wkly. Rep.* **70**, 1337–1343 (2021). doi:10.15585/mmwr.mm7038e1 Medline
16. Y. Goldberg *et al.*, Waning immunity of the BNT162b2 vaccine: A nationwide study from Israel. *medRxiv*, (2021). doi:10.1101/2021.08.24.21262423.
17. N. Dagan, N. Barda, E. Kepten, O. Miron, S. Perchik, M. A. Katz, M. A. Hernán, M. Lipsitch, B. Reis, R. D. Balicer, BNT162b2 mRNA Covid-19 Vaccine in a Nationwide Mass Vaccination Setting. *N. Engl. J. Med.* **384**, 1412–1423 (2021). doi:10.1056/NEJMoa2101765 Medline
18. "Israel expands COVID vaccine booster campaign to over 50s, health workers., *Reuters*, 13 August 2021.
19. Y. M. Bar-On, Y. Goldberg, M. Mandel, O. Bodenheimer, L. Freedman, N. Kalkstein, B. Mizrahi, S. Alroy-Preis, N. Ash, R. Milo, A. Huppert, Protection of BNT162b2 Vaccine Booster against Covid-19 in Israel. *N. Engl. J. Med.* **385**, 1393–1400 (2021). doi:10.1056/NEJMoa2114255 Medline
20. J. Lopez Bernal, N. Andrews, C. Gower, E. Gallagher, R. Simmons, S. Thelwall, J. Stowe, E. Tessier, N. Groves, G. Dabrera, R. Myers, C. N. J. Campbell, G. Amirthalingam, M. Edmunds, M. Zambon, K. E. Brown, S. Hopkins, M. Chand, M. Ramsay, Effectiveness of Covid-19 Vaccines against the B.1.617.2 (Delta) Variant. *N. Engl. J. Med.* **385**, 585–594 (2021). doi:10.1056/NEJMoa2108891 Medline
21. K. B. Pouwels *et al.*, Impact of Delta on viral burden and vaccine effectiveness against new SARS-CoV-2 infections in the UK. *medRxiv*, 2021.2008.2018.21262237 (2021). doi:10.1101/2021.08.18.21262237.
22. FDA, "FDA Authorizes Booster Dose of Pfizer-BioNTech COVID-19 Vaccine for Certain Populations" (www.fda.gov/news-events/press-announcements/fda-authorizes-booster-dose-pfizer-biontech-covid-19-vaccine-certain-populations).
23. FDA, "Coronavirus (COVID-19) Update: FDA Takes Additional Actions on the Use of a Booster Dose for COVID-19 Vaccines" (www.fda.gov/news-events/press-announcements/coronavirus-covid-19-update-fda-takes-additional-actions-use-booster-dose-covid-19-vaccines).
24. P. R. Krause, T. R. Fleming, R. Peto, I. M. Longini, J. P. Figueroa, J. A. C. Sterne, A. Cravioto, H. Rees, J. P. T. Higgins, I. Boutron, H. Pan, M. F. Gruber, N. Arora, F. Kazi, R. Gaspar, S. Swaminathan, M. J. Ryan, A.-M. Henao-Restrepo, Considerations in boosting COVID-19 vaccine immune responses. *Lancet* **398**, 1377–1380 (2021). doi:10.1016/S0140-6736(21)02046-8 Medline
25. CDC COVID-19 Vaccine Breakthrough Case Investigations Team, COVID-19 Vaccine Breakthrough Infections Reported to CDC - United States, January 1-April 30, 2021. *MMWR Morb. Mortal. Wkly. Rep.* **70**, 792–793 (2021). Medline
26. N. Brusselaers, J. Lagergren, The Charlson Comorbidity Index in Registry-based Research. *Methods Inf. Med.* **56**, 401–406 (2017). doi:10.3414/ME17-01-0051 Medline
27. Y. Young-Xu, C. Korves, J. Roberts, E. I. Powell, G. M. Zwain, J. Smith, H. S. Izurieta, Coverage and Estimated Effectiveness of mRNA COVID-19 Vaccines Among US Veterans. *JAMA Netw. Open* **4**, e2128391 (2021). doi:10.1001/jamanetworkopen.2021.28391 Medline
28. Y. Wang, R. Chen, F. Hu, Y. Lan, Z. Yang, C. Zhan, J. Shi, X. Deng, M. Jiang, S. Zhong, B. Liao, K. Deng, J. Tang, L. Guo, M. Jiang, Q. Fan, M. Li, J. Liu, Y. Shi, X. Deng, X. Xiao, M. Kang, Y. Li, W. Guan, Y. Li, S. Li, F. Li, N. Zhong, X. Tang, Transmission, viral kinetics and clinical characteristics of the emergent SARS-CoV-2 Delta VOC in Guangzhou, China. *EClinicalMedicine* **40**, 101129 (2021). doi:10.1016/j.eclinm.2021.101129 Medline
29. K. Bruxvoort *et al.*, Effectiveness of mRNA-1273 against Delta, Mu, and other emerging variants. *medRxiv*, (2021). doi:10.1101/2021.09.29.21264199.
30. A. Puranik *et al.*, Comparison of two highly-effective mRNA vaccines for COVID-19 during periods of Alpha and Delta variant prevalence. *medRxiv*, (2021). doi:10.1101/2021.08.06.21261707.
31. S. Y. Tartof, J. M. Slezak, H. Fischer, V. Hong, B. K. Ackerson, O. N. Ranasinghe, T. B. Frankland, O. A. Ogun, J. M. Zamparo, S. Gray, S. R. Valluri, K. Pan, F. J. Angulo, L. Jodar, J. M. McLaughlin, Effectiveness of mRNA BNT162b2 COVID-19 vaccine up to 6 months in a large integrated health system in the USA: A retrospective cohort study. *Lancet* **398**, 1407–1416 (2021). doi:10.1016/S0140-6736(21)02183-8 Medline
32. H. Chemaitelly, P. Tang, M. R. Hasan, S. AlMukdad, H. M. Yassine, F. M. Benslimane, H. A. Al Khatib, P. Coyle, H. H. Ayoub, Z. Al Kanaani, E. Al Kuwari, A.

Jeremijenko, A. H. Kaleeckal, A. N. Latif, R. M. Shaik, H. F. Abdul Rahim, G. K. Nasrallah, M. G. Al Kuwari, H. E. Al Romaihi, A. A. Butt, M. H. Al-Thani, A. Al Khal, R. Bertollini, L. J. Abu-Raddad, Waning of BNT162b2 Vaccine Protection against SARS-CoV-2 Infection in Qatar. *N. Engl. J. Med.* 10.1056/NEJMoa2114114 (2021). doi:10.1056/NEJMoa2114114 Medline

33. H. Chemaitelly, H. M. Yassine, F. M. Benslimane, H. A. Al Khatib, P. Tang, M. R. Hasan, J. A. Malek, P. Coyle, H. H. Ayoub, Z. Al Kanaani, E. Al Kuwari, A. Jeremijenko, A. H. Kaleeckal, A. N. Latif, R. M. Shaik, H. F. Abdul Rahim, G. K. Nasrallah, M. G. Al Kuwari, H. E. Al Romaihi, M. H. Al-Thani, A. Al Khal, A. A. Butt, R. Bertollini, L. J. Abu-Raddad, mRNA-1273 COVID-19 vaccine effectiveness against the B.1.1.7 and B.1.351 variants and severe COVID-19 disease in Qatar. *Nat. Med.* **27**, 1614–1621 (2021). doi:10.1038/s41591-021-01446-y Medline
34. T. Tada *et al.*, Comparison of Neutralizing Antibody Titers Elicited by mRNA and Adenoviral Vector Vaccine against SARS-CoV-2 Variants. *bioRxiv*, 2021.2007.2019.452771 (2021). doi:10.1101/2021.07.19.452771.
35. R. R. Goel, M. M. Painter, S. A. Apostolidis, D. Mathew, W. Meng, A. M. Rosenfeld, K. A. Lundgreen, A. Reynaldi, D. S. Khoury, A. Pattekar, S. Gouma, L. Kuri-Cervantes, P. Hicks, S. Dysinger, A. Hicks, H. Sharma, S. Herring, S. Korte, A. E. Baxter, D. A. Oldridge, J. R. Giles, M. E. Weirick, C. M. McAllister, M. Awofolaju, N. Tanenbaum, E. M. Drapeau, J. Dougherty, S. Long, K. D'Andrea, J. T. Hamilton, M. McLaughlin, J. C. Williams, S. Adamski, O. Kuthuru, I. Frank, M. R. Betts, L. A. Vella, A. Grifoni, D. Weiskopf, A. Sette, S. E. Hensley, M. P. Davenport, P. Bates, E. T. Luning Prak, A. R. Greenplate, E. J. Wherry, S. Adamski, Z. Alam, M. M. Addison, K. T. Byrne, A. Chandra, H. C. Descamps, N. Han, Y. Kaminskiy, S. C. Kammerman, J. Kim, A. R. Greenplate, J. T. Hamilton, N. Markosyan, J. H. Noll, D. K. Omran, A. Pattekar, E. Perkey, E. M. Prager, D. Pueschl, A. Rennels, J. B. Shah, J. S. Shilan, N. Wilhausen, A. N. Vanderbeck; UPenn COVID Processing Unit‡, mRNA vaccines induce durable immune memory to SARS-CoV-2 and variants of concern. *Science* 10.1126/science.abm0829 (2021). doi:10.1126/science.abm0829 Medline
36. K. B. Pouwels, E. Pritchard, P. C. Matthews, N. Stoesser, D. W. Eyre, K.-D. Vihta, T. House, J. Hay, J. I. Bell, J. N. Newton, J. Farrar, D. Crook, D. Cook, E. Rourke, R. Studley, T. E. A. Peto, I. Diamond, A. S. Walker, Effect of Delta variant on viral burden and vaccine effectiveness against new SARS-CoV-2 infections in the UK. *Nat. Med.* 10.1038/s41591-021-01548-7 (2021). doi:10.1038/s41591-021-01548-7 Medline
37. R. M. Anderson, C. Vegvari, T. D. Hollingsworth, L. Pi, R. Maddren, C. W. Ng, R. F. Baggaley, The SARS-CoV-2 pandemic: Remaining uncertainties in our understanding of the epidemiology and transmission dynamics of the virus, and challenges to be overcome. *Interface Focus* **11**, 20210008 (2021). doi:10.1098/rsfs.2021.0008
38. D. W. Eyre *et al.*, The impact of SARS-CoV-2 vaccination on Alpha & Delta variant transmission. *medRxiv*, 2021.2009.2028.21264260 (2021). doi:10.1101/2021.09.28.21264260.
39. M. J. M. Niesen *et al.*, COVID-19 vaccines dampen genomic diversity of SARS-CoV-2: Unvaccinated patients exhibit more antigenic mutational variance. *medRxiv*, 2021.2007.2001.21259833 (2021). doi:10.1101/2021.07.01.21259833.
40. C. H. Sudre, B. Murray, T. Varsavsky, M. S. Graham, R. S. Penfold, R. C. Bowyer, J. C. Pujol, K. Klaser, M. Antonelli, L. S. Canas, E. Molteni, M. Modat, M. Jorge Cardoso, A. May, S. Ganesh, R. Davies, L. H. Nguyen, D. A. Drew, C. M. Astley, A. D. Joshi, J. Merino, N. Tsereteli, T. Fall, M. F. Gomez, E. L. Duncan, C. Menni, F. M. K. Williams, P. W. Franks, A. T. Chan, J. Wolf, S. Ourselin, T. Spector, C. J. Steves, Attributes and predictors of long COVID. *Nat. Med.* **27**, 626–631 (2021). doi:10.1038/s41591-021-01292-y Medline
41. B. van den Borst, Recovery after Covid-19. *Lancet Reg. Health West. Pac.* **12**, 100208 (2021). doi:10.1016/j.lanwpc.2021.100208 Medline
42. J. L. Hirschtick, A. R. Titus, E. Slocum, L. E. Power, R. E. Hirschtick, M. R. Elliott, P. McKane, N. L. Fleischer, Population-based estimates of post-acute sequelae of SARS-CoV-2 infection (PASC) prevalence and characteristics. *Clin. Infect. Dis.* 10.1093/cid/ciab408 (2021). Medline
43. Pfizer, "Pfizer and BioNTech Announce phase 3 trial data showing high efficacy of a booster dose of their COVID-19 vaccine," press release 21 October 2021; www.pfizer.com/news/press-release/press-release-detail/pfizer-and-biontech-announce-phase-3-trial-data-showing.
44. L. SAAD., Americans Getting Out More, but Cautiously (2021). Accessed October 22, 2021. https://news.gallup.com/poll/350666/americans-getting-cautiously.aspx
45. K. L. Bajema, R. M. Dahl, M. M. Prill, E. Meites, M. C. Rodriguez-Barradas, V. C. Marconi, D. O. Beenhouwer, S. T. Brown, M. Holodniy, C. Lucero-Obusan, G. Rivera-Dominguez, R. G. Morones, A. Whitmire, E. B. Goldin, S. L. Evener, M. Tremarelli, S. Tong, A. J. Hall, S. J. Schrag, M. McMorrow, M. Kobayashi, J. R. Verani, D. Surie, G. Ahmadi-Izadi, J. Burnette, R. Deovic, L. Epstein, A. Hartley, E. Morales, T. Tanner, N. Patel, A. Tunson, K. Elliot, I. Graham, D. Lama, I. Pena, A. Perea, G. A. Perez, J. Simelane, S. Smith, G. Tallin, A. Tisi, A. A. Lopez, M. C. Gonzalez, B. Lengi, D. Mansouri, M. V. Tamez, B. Aryanfar, I. Lee-Chang, C. Jeong, A. Matolek, C. Mendoza, A. Poteshkina, S. Naeem, M. Agrawal, J. Lopez, T. Peters, G. Kudryavtseva, J. Cates, J. M. Folster, A. Kambhampati, A. Kelleher, Y. Li, H. J. Ng, Y. Tao; SUPERNOVA COVID-19; Surveillance Group; Surveillance Platform for Enteric and Respiratory Infectious Organisms at the VA (SUPERNOVA) COVID-19 Surveillance Group, Effectiveness of COVID-19 mRNA Vaccines Against COVID-19-Associated Hospitalization - Five Veterans Affairs Medical Centers, United States, February 1-August 6, 2021. *MMWR Morb. Mortal. Wkly. Rep.* **70**, 1294–1299 (2021). doi:10.15585/mmwr.mm7037e3 Medline
46. P. Cirillo, N. Krigbaum, Code for Methods for SARS-CoV-2 Vaccine Protection and Deaths among U.S. Veterans during 2021. Zenodo (2021); doi:10.5281/zenodo.5609444.

ACKNOWLEDGMENTS

We acknowledge the invaluable efforts of the Veterans Affairs data architects, managers, and clinicians who assembled the Centralized Interactive Phenomics Resource (CIPHER), rapidly compiling a library of numerous COVID-19-related phenotypes that are the basis for this research. Our work was supported using resources and facilities of the Department of Veterans Affairs (VA) Informatics and Computing Infrastructure (VINCI), VA HSR RES 14-457. We deeply appreciate the steady service and support of the VA Informatics and Computing Infrastructure (VINCI) staff. Without the efforts of these teams, this study would not have been possible. We are grateful for the Veterans who have so selflessly served their country. The views expressed in this article are those of the authors and do not necessarily reflect the position or policy of the Department of Veterans Affairs or the United States government. IRB: This research is covered under the University of California, San Francisco IRB # 10-03609 Reference #: 320151 **Funding**: Mercatus Center at George Mason University (Fast Grants #2207); University of California Office of the President (Emergency COVID-19 Research Seed Funding R00RG3118). **Author contributions:** Conceptualization: BAC, PMC Methodology: PMC, CCM, BAC Statistical analysis: PMC Funding acquisition: AWW, PMC, NYK Data interpretation: all authors Writing - original draft: CCM, PMC, BAC Writing - review and editing: all authors. **Competing interests**: CCM reports consulting for Freenome. AWW reports consulting for ECOM Medical, Obelab, Sensifree, and Shifamed. BAC, PMC, and NYK declare that they have no competing interests. **Data and materials availabilit**y: Data and materials availability: The data that support the findings of this study are available from the Department of Veterans Affairs (VA). Code is available at DOI: 10.5281/zenodo.5609444 (*46*). Data are made freely available to researchers behind the VA firewall with an approved study protocol. Summary data can be accessed from a commercial source Data Lake Analysis for Real-World Evidence Solutions – STATinMED: https://statinmed.com/data. More information is available at https://www.virec.research.va.gov or by contacting the VA Information Resource Center at VIReC@va.gov. This work is licensed under a Creative Commons Attribution 4.0 International (CC BY 4.0) license, which permits unrestricted use, distribution, and reproduction in any medium, provided the original work is properly cited. To view a copy of this license, visit https://creativecommons.org/licenses/by/4.0/. This license does not apply to figures/photos/artwork or other content included in the article that is credited to a third party; obtain authorization from the rights holder before using such material.

SUPPLEMENTARY MATERIALS

science.org/doi/10.1126/science.abm0620
Material and Methods
Fig. S1
Tables S1 to S3
MDAR Reproducibility Checklist

24 August 2021; accepted 2 November 2021
Published online 4 November 2021
10.1126/science.abm0620

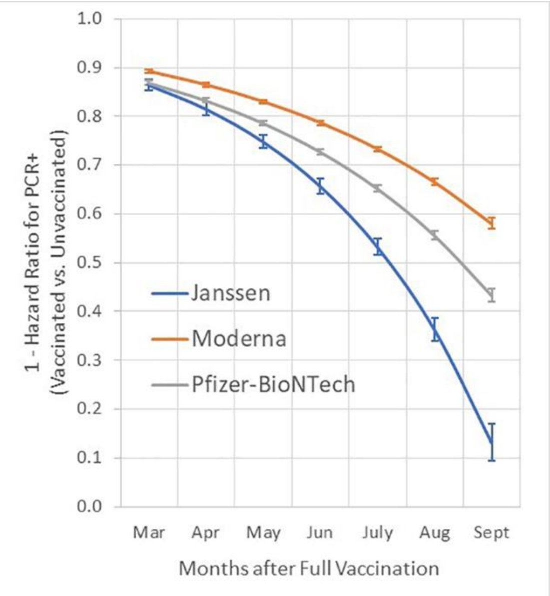

Fig. 1. Time dependent vaccine effectiveness against SARS-CoV-2 infection as estimated from Cox proportional hazards models, adjusted for age, race, ethnicity, sex, and comorbidity score. Vaccine effectiveness presented as (1 − hazard ratio × 100) and 95% confidence intervals. Effectiveness for each month was estimated from contrasts using product terms for vaccination status by time to most recent RT-PCR assay.

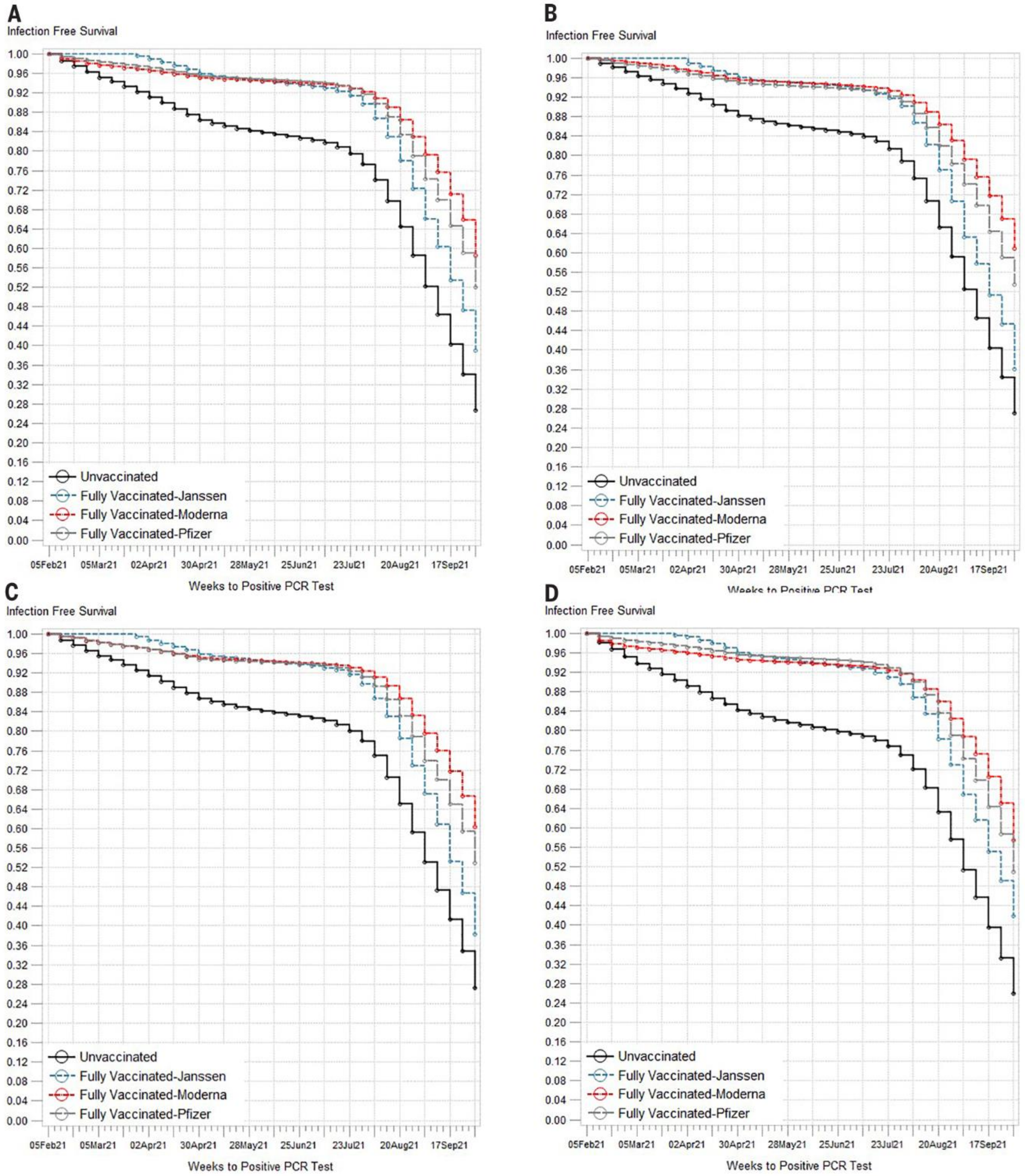

Fig. 2. Kaplan-Meier curves illustrating cumulative risk of SARS-CoV-2 infection by vaccination status and age. (**A**) All ages. (**B**) Age <50 years. (**C**) Age 50-64 years. (**D**) Age ≥65 years. The survival function estimates time to infection detected by most recent RT-PCR assay.

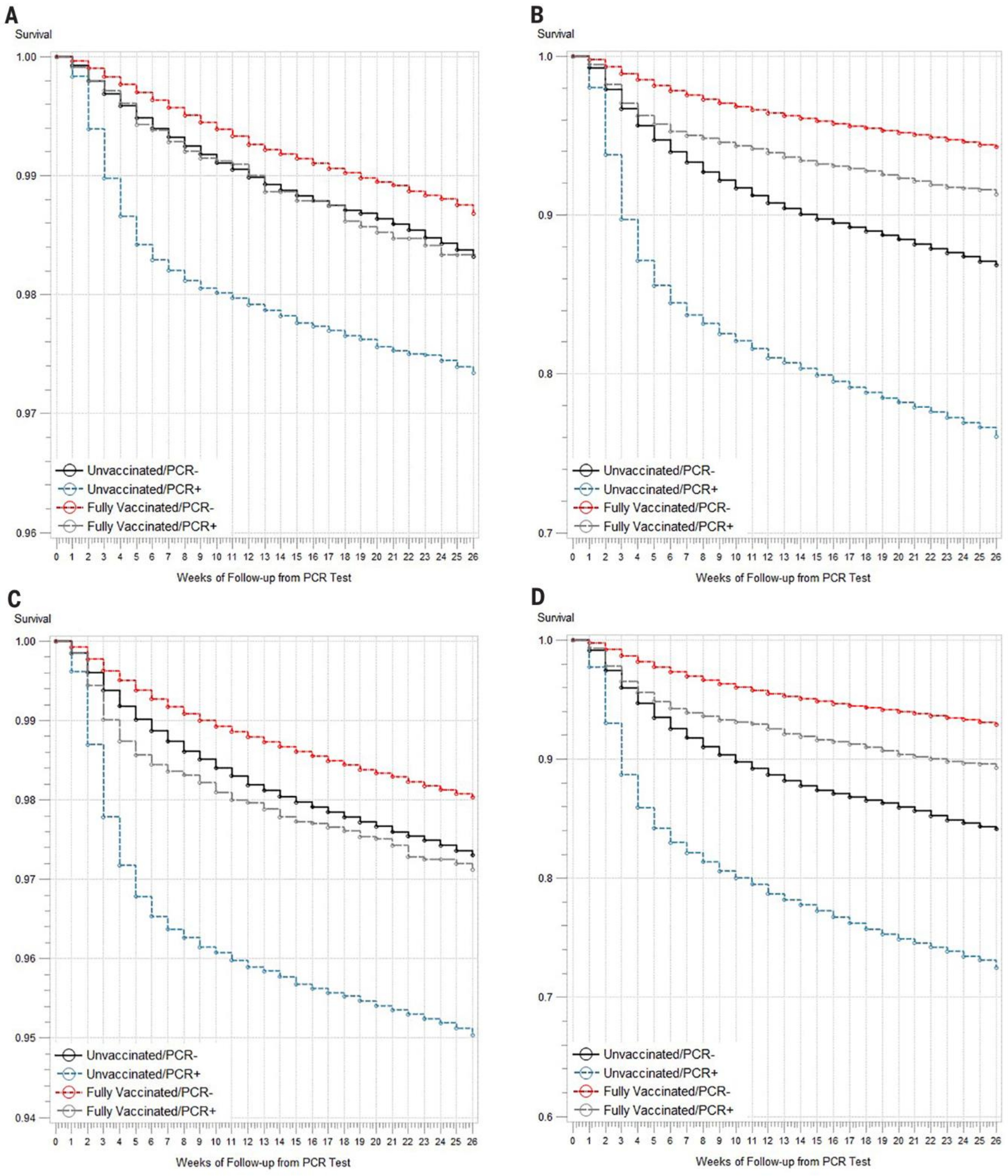

Fig. 3. Kaplan-Meier curves illustrating cumulative risk of death due to any cause by vaccination status and RT-PCR assay. (**A**) Age <65 years. (**B**) Age ≥65 years. (**C**) Charlson Comorbidity Index score <3. (**D**) Charlson Comorbidity Index score ≥3.

Table 1. Vaccine effectiveness against SARS-CoV-2 infection* by month after vaccination†; estimated from Cox proportional hazards models‡; and adjusted for age, race, ethnicity, sex, and comorbidity score.

	Adjusted hazard ratio	95% confidence interval		*P* value
Janssen versus unvaccinated§				
March	0.14	0.12	0.15	<0.01
April	0.19	0.17	0.20	<0.01
May	0.25	0.24	0.27	<0.01
June	0.34	0.33	0.36	<0.01
July	0.47	0.45	0.49	<0.01
August	0.64	0.62	0.66	<0.01
September	0.87	0.83	0.91	<0.01
Moderna versus unvaccinated§				
March	0.11	0.10	0.11	<0.01
April	0.14	0.13	0.14	<0.01
May	0.17	0.17	0.17	<0.01
June	0.21	0.21	0.22	<0.01
July	0.27	0.26	0.27	<0.01
August	0.33	0.33	0.34	<0.01
September	0.42	0.41	0.43	<0.01
Pfizer-BioNTech versus unvaccinated§				
March	0.13	0.13	0.14	<0.01
April	0.17	0.16	0.17	<0.01
May	0.21	0.21	0.22	<0.01
June	0.27	0.27	0.28	<0.01
July	0.35	0.34	0.35	<0.01
August	0.44	0.44	0.45	<0.01
September	0.57	0.55	0.58	<0.01

*Adjusted hazard ratio < 1.0 indicates lower risk of infection for vaccine, shown compared to unvaccinated. For vaccinated Veterans, infection is assessed 15 days after the last vaccine that established full vaccination status. For unvaccinated Veterans, infection is assessed beginning in February 1, 2021, coincident with broadscale vaccine eligibility in the VA.

†Time dependence was tested in Cox proportional hazards models by including product terms for vaccination status (Janssen, Moderna, Pfizer-BioNTech, unvaccinated) by log(time): Janssen*log(time), Moderna*log(time), Pfizer-BioNTech*log(time); and adjusted for age, sex, race, ethnicity, and comorbidity (Charlson Comorbidity score, overweight, type II diabetes, chronic obstructive pulmonary disease, bronchitis, acute respiratory failure, and chronic lung disease). Significance levels for all product terms were $P < 0.0001$.

‡Vaccination status is modeled as time-varying, assigning follow-up time for Veterans before the date of full vaccination as unvaccinated time and time after the date of full vaccination as vaccinated time; vaccination is defined as: 1) a single Janssen vaccine; 2) two Moderna vaccines; or 3) two Pfizer-BioNTech vaccines.

§Associations at each month were estimated from contrasts using product terms for vaccination status by time in Cox proportional hazards models, including indicator terms for vaccination status (Janssen, Moderna, Pfizer-BioNTech) product terms, and age, sex, race, ethnicity, and comorbidity (Charlson Comorbidity score, overweight, Type II diabetes, chronic obstructive pulmonary disease, bronchitis, acute respiratory failure, and chronic lung disease).
