# Supplementary material for: SARS-CoV-2 vaccine protection and deaths among US veterans during 2021
Source: Science. 2021 Nov 4;375(6578):331–6. doi: 10.1126/science.abm0620 (PMC9836205; doi:10.1126/science.abm0620)

Science
AAAS

Supplementary Materials for

SARS-CoV-2 vaccine protection and deaths among US veterans during 2021

Barbara A. Cohn *et al.*

Corresponding author: Arthur W. Wallace, art.wallace@va.gov

Science **375**, 331 (2022)
DOI: 10.1126/science.abm0620

The PDF file includes:

Material and Methods
Fig. S1
Tables S1 to S3

Other Supplementary Material for this manuscript includes the following:

MDAR Reproducibility Checklist

Materials and Methods

We examined SARS CoV-2 infections and death due to any cause in U.S. Veterans age ≥18 years and receiving care in the Veterans Health Administration (VHA), the largest integrated health system in the country. After the U.S. Food and Drug Administration issued an emergency use authorization for the Pfizer-BioNTech vaccine in December 2020, the Department of Veterans Affairs (VA) first provided vaccinations to front-line health care workers and Veterans residing in long-term care facilities in 37 of its medical centers across the U.S. As vaccine supplies increased, additional Veterans received vaccinations based on age, existing health conditions, and other factors for increased risk of severe illness or death from COVID-19, and as of October 21, 2021, more than 3.5 million Veterans have been fully vaccinated (available at: https://www.accesstocare.va.gov/Healthcare/COVID19NationalSummary).

We used the VA Corporate Data Warehouse (CDW) to identify vaccination status (fully vaccinated vs. unvaccinated), vaccine type (Pfizer-BioNTech, Moderna, Janssen), SARS CoV-2 infections, and deaths due to any cause during the period February 1, 2021 to October 1, 2021.We focused on this time period because it encompasses the time when many Veterans became fully vaccinated (i.e., vaccine eligibility extended beyond long-term care facilities) and the mid-summer 2021 surge in cases in the U.S. The VA CDW provides discrete, individual-level data, including demographics, administrative claims-based diagnosis and procedure codes, prescriptions, anthropometric measures, and free-text data including procedure notes and pathology reports; data include all 50 states and U.S. territories.

Fully vaccinated was defined as two doses of Pfizer-BioNTech or Moderna or one dose of Janssen vaccines, administered at the appropriate intervals. We excluded partial vaccinations and vaccinations that were administered off-label and/or not according to recommendation. We did not consider booster vaccines as the study period largely precedes authorization for boosters in the U.S.

SARS-CoV-2 infection was defined as the detection of SARS-CoV-2 on most recent reverse-transcriptase–polymerase-chain-reaction (RT-PCR) assay, regardless of symptoms or test setting. The reason for RT-PCR assay is not provided in the VA CDW. Veterans may have received a RT-PCR assay for many reasons, including but not limited to, concern about exposure, symptoms, as a requirement of a clinic visit or in advanced of a medical procedure, or as part of a hospital admission. We included assays received for any reason or in any clinical setting.

Deaths due to any cause (n=22,345) were identified using a combination of data from the Master Veteran Index, vital status files, and medical records (hierarchical in that order). These data include deaths that occurred both inside and outside the VHA.

Vaccine effectiveness (1- adjusted hazard ratio [aHR] x 100) against infection was estimated using Cox proportional hazards models, overall and by vaccine type (Pfizer-BioNTech vs. Moderna vs. Janssen), with vaccination status modeled as time-varying. Modeling vaccination as time-varying assigns follow-up time for Veterans before the date of full vaccination (defined as 14 days after receipt of the second dose of Pfizer-BioNTech or Moderna or one dose of Janssen vaccines) as unvaccinated time and time after the date of full vaccination as vaccinated time; those never vaccinated contribute only unvaccinated time We required Veterans to receive a RT-PCR assay to contribute vaccinated and/or unvaccinated follow-up time, such that Veterans who received a recent RT-PCR assay before vaccination contributed unvaccinated time only. Veterans in both groups were followed from February 1, 2021 until their most recent RT-PCR assay or October 1, 2021. We used calendar time as the underlying time scale to allow the baseline hazard to vary flexibly as vaccine eligibility, testing practices, non-pharmaceutical interventions, and infection transmission changed over time. As noted by others (31), models with calendar time as the underlying time scale compare those who are unvaccinated on each calendar date to those who are vaccinated on that same date.

We report aHR and 95% confidence intervals (CI), adjusted for age, sex and comorbidity. Comorbidity was measured using the Charlson comorbidity index (*26*), and a diagnosis of diabetes, chronic obstructive pulmonary disease, bronchitis, acute respiratory failure, chronic lung disease, cardiovascular disease in the two years prior to the RT-PCR assay. We also examined time dependence by including product terms for vaccination status by the log of follow-up time, with p<0.01 indicating statistical significance.

To illustrate findings, we plotted cumulative risk of infection using Kaplan-Meier estimation to account for censoring, overall and by vaccination status and age group (<50 years, 50-64 years, and ≥65 years). We used the same time-varying assignment described above allowing individuals to contribute time before and after vaccination and allowing for changes in RT-PCR status. Thus, as described above for Cox models, the time scale compares those who are unvaccinated on each calendar date to those who are vaccinated on that same date. We selected these age groups because they correspond to the phased-in eligibility for vaccination.

We examined vaccine effectiveness against death in a nested sample (n=775,536) of Veterans who were: 1) unvaccinated and received an RT-PCR assay from February 1, 2021 to September 30, 2021; or 2) fully vaccinated and received an RT-PCR assay after the date of full vaccination during the same period. We plotted cumulative risk of death due to any cause using Kaplan-Meier estimation, separately by age group <65 vs. ≥65 years) and comorbidity score (Charlson Comorbidity Index, <3 vs. ≥3) (*26*). Follow-up was accrued from the date of RT-PCR assay until death or October 1, 2021. In addition, we used Cox proportional hazards models to examine vaccine effectiveness against death during the period corresponding to the emergence and dominance of the Delta variant in the U.S. We limited the nested sample for this analysis to RT-PCR assays on or after July 1, 2021 (n=312,829) and report aHR and 95% CI.

All analyses were conducted using SAS Enterprise Guide 7.1 (SAS Institute, Cary, NC). This study was approved by the Institutional Review Board at the University of California San Francisco and the Public Health Institute, as well as the San Francisco VA Research and Development Committee.

Supplementary Table 1. Distribution of SARS-CoV-2 infection by demographics and vaccination status in 780,225 U.S. Veterans, February 1, 2021 to October 1, 2021

		Most recent RT-PCR[1]	
		Negative	Positive (%)
Vaccination status[2]			
	Unvaccinated	209,439	72,638 (25.8%)
	Janssen	32,271	3,391 (9.5%)
	Moderna	217,207	13,555 (5.9%)
	Pfizer-BioNTech	215,156	16,568 (7.2%)
Sex			
	Female	94,315	17,644 (15.8%)
	Male	579,758	88,508 (13.2%)
Ethnicity			
	Hispanic	51,483	8,320 (13.9%)
	Non-Hispanic	622,590	97,832 (13.6%)
Race			
	American Indian/Alaska Native	5,091	880 (14.7%)
	Asian	7,109	854 (10.7%)
	Black or African American	148,967	19,512 (11.6%)
	Native Hawaiian/Other Pacific Islander	5,807	962 (14.2%)
	White	437,860	70,198 (13.8%)
Age at RT-PCR (years)			
	<50	149,961	35,476 (19.1%)
	50-64	193,567	29,419 (13.2%)
	≥65	330,545	41,257 (11.1%)
Comorbidity score[3] (Charlson Comorbidity Index)			
	0	263,218	50,941 (16.2%)
	1-2	227,305	32,332 (12.5%)
	3-4	106,331	13,029 (10.9%)
	≥5	77,219	9,850 (11.3%)

[1]For vaccinated Veterans, RT-PCR assessed 15 days after last dose that established full vaccination status; for unvaccinated Veterans, RT-PCR assessed beginning in February 1, 2021, coincident with broadscale vaccine eligibility in the VA. [2]Vaccination status defined as: 1) a single Janssen vaccine; 2) two Moderna vaccines or 3) two Pfizer-BioNTech vaccines.[3]Pre-existing morbidity as represented by the Charlson Co-Morbidity Index reported within 2 years of first RT-PCR test or hospitalization related to first RT-PCR test.

Supplementary Table 2. Distribution of vaccine type by demographics among 498,148 fully vaccinated U.S. Veterans, February 1, 2021 to October 1, 2021

	Vaccine Type[1]		
	Janssen (%)	Moderna (%)	Pfizer (%)
Total Vaccinated	35,662 (7.2%)	230,762 (46.3%)	231,724 (46.5%)
February	30 (0.0%)	32,114 (41.3%)	45,675 (58.7%)
March	5,862 (3.6%)	86,107 (52.2%)	72,828 (44.2%)
April	10,732 (7.6%)	65,530 (46.1%)	65,852 (46.3%)
May	4,057 (7.0%)	27,986 (48.5%)	25,651 (44.5%)
June	5,844 (24.0%)	9,176 (37.7%)	9,305 (38.3%)
July	3,358 (29.6%)	3,694 (32.5%)	4,305 (37.9%)
August	3,645 (37.4%)	2,491 (25.5%)	3,621 (37.1%)
September	2,143 (20.8%)	3,664 (35.6%)	4,487 (43.6%)
Sex			
Female	3,884 (8.1%)	19,752 (41.2%)	24,295 (50.7%)
Male	31,778 (7.1%)	211,010 (46.9%)	207,429 (46.1%)
Ethnicity			
Hispanic	2,703 (6.7%)	19,659 (48.9%)	17,842 (44.4%)
Non-Hispanic	32,959 (7.2%)	211,103 (46.1%)	213,882 (46.7%)
Race			
American Indian/Alaska Native	316 (8.5%)	1,746 (47.0%)	1,657 (44.6%)
Asian	349 (6.4%)	2,382 (43.4%)	2,753 (50.2%)
Black or African American	7,499 (6.4%)	44,476 (38.2%)	64,425 (55.4%)
Native Hawaiian/Other Pacific Islander	295 (6.7%)	1,973 (44.8%)	2,131 (48.4%)
White	24,900 (7.4%)	166,448 (49.3%)	146,199 (43.3%)
Age (years)			
<50	8,855 (12.8%)	26,253 (38.0%)	33,981 (49.2%)
50-64	13,823 (10.0%)	59,240 (43.0%)	64,644 (46.9%)
≥65	12,984 (4.5%)	145,269 (49.9%)	291,352 (58.5%)

[1]Vaccination status defined as: 1) a single Janssen vaccine; 2) two Moderna vaccines or 3) two Pfizer-BioNTech vaccines

Supplementary Table 3A: RT-PCR Assays by Vaccination Status and Week, February 1 – October 1, 2021 (used for Figure 2A): All Ages

	Unvaccinated		Vaccinated					
			Janssen		Moderna		Pfizer-BioNTech	
Week	PCR+	PCR-	PCR+	PCR-	PCR+	PCR-	PCR+	PCR-
1	6543	17817	0	0	31	83	24	113
2	4557	16543	0	0	32	171	45	329
3	5132	20736	0	1	86	650	124	866
4	4548	18848	0	0	111	1062	146	1448
5	3176	18010	0	0	109	1402	179	1978
6	3639	16699	1	6	180	1992	236	2536
7	3739	16623	9	79	261	2611	272	2935
8	3615	14775	29	137	293	3221	330	3297
9	3607	14534	38	189	348	3853	391	4015
10	3619	13969	55	259	408	4555	485	4498
11	3231	13401	67	369	454	5009	530	5029
12	2915	11258	104	406	475	5481	569	5609
13	1608	9855	50	458	176	5513	196	5606
14	1388	9350	26	425	158	5762	183	5900
15	1178	8425	27	458	173	5920	153	6018
16	968	7427	29	462	147	5491	133	5605
17	772	6884	32	449	150	5498	147	5563
18	754	7689	30	583	135	6312	123	6384
19	675	7178	31	553	162	5984	130	6368
20	699	7284	32	626	129	6320	144	6166
21	703	6663	34	591	136	5788	145	5685
22	835	6681	39	613	150	5982	180	5831
23	1378	8265	72	794	230	7215	376	6822
24	1989	9216	91	841	371	7592	503	7187
25	2923	10112	158	874	471	7860	711	7563
26	3840	11371	258	996	756	8699	1077	8410
27	4621	11740	285	1068	896	8942	1268	8792
28	4721	6532	321	629	989	4980	1353	4568
29	4632	5112	333	525	1149	3752	1378	3252
30	4488	4574	330	478	1054	3477	1305	3012
31	3486	4015	263	439	855	3344	1009	2774
32	3136	4494	268	481	880	3696	1037	3221
33	2452	4154	180	465	758	3536	803	3081
34	1966	3993	162	462	649	3280	646	2937

NOTE: Because vaccination status was modeled as a time-varying covariate (see Materials and Methods), Veterans could contribute both vaccinated and unvaccinated time. Therefore, the total number of observations in the table does not represent the unique number of Veterans.

Supplementary Table 3B: RT-PCR Assays by Vaccination Status and Week, February 1 – October 1, 2021 (used for Figure 2B): Age <50 years

		Unvaccinated			Vaccinated							
					Janssen			Moderna			Pfizer-BioNTech	
Week		PCR+	PCR-		PCR+	PCR-		PCR+	PCR-		PCR+	PCR-
1		1598	4162		0	0		3	21		2	12
2		1136	3582		0	0		3	28		5	16
3		1316	4542		0	0		6	53		6	48
4		1266	4098		0	0		6	53		9	70
5		995	4016		0	0		9	81		8	86
6		1093	4006		0	1		10	97		13	93
7		1236	4183		1	11		23	135		18	122
8		1241	4151		9	20		18	154		25	149
9		1337	4174		6	37		20	168		23	212
10		1335	4357		11	39		33	243		43	286
11		1289	4487		12	71		43	307		35	365
12		1053	3929		18	93		28	344		63	485
13		627	3520		11	104		10	397		14	546
14		488	3447		5	83		11	475		20	575
15		411	2934		3	90		14	493		18	643
16		343	2672		2	84		16	427		19	599
17		279	2406		4	95		10	489		23	620
18		262	2691		5	134		10	524		12	733
19		240	2488		6	117		17	513		20	703
20		263	2559		7	117		16	535		23	687
21		296	2313		11	115		19	502		19	605
22		371	2437		9	118		18	521		23	716
23		631	3127		19	165		23	709		55	852
24		974	3742		21	167		48	720		77	896
25		1458	4186		36	199		65	805		106	1068
26		1870	5025		73	242		104	1002		210	1256
27		2153	5315		84	267		112	1086		204	1251
28		2104	2966		84	149		117	591		218	718
29		2082	2311		94	132		128	491		180	527
30		2032	2089		96	129		129	435		179	522
31		1528	1805		61	104		100	419		157	451
32		1342	2012		61	109		86	454		157	526
33		987	1833		43	115		76	433		115	478
34		798	1639		46	100		59	389		71	469

NOTE: Because vaccination status was modeled as a time-varying covariate (see Materials and Methods), Veterans could contribute both vaccinated and unvaccinated time. Therefore, the total number of observations in the table does not represent the unique number of Veterans.

Supplementary Table 3C: RT-PCR Assays by Vaccination Status and Week, February 1 – October 1, 2021 (used for Figure 2C): Age 50-64 years

	Unvaccinated		Vaccinated					
			Janssen		Moderna		Pfizer-BioNTech	
Week	PCR+	PCR-	PCR+	PCR-	PCR+	PCR-	PCR+	PCR-
1	1838	5223	0	0	9	24	5	21
2	1368	4621	0	0	8	49	4	57
3	1501	5677	0	0	24	124	23	125
4	1377	5360	0	0	23	177	29	183
5	945	5335	0	0	22	209	34	293
6	1172	5165	1	3	31	275	32	340
7	1218	5277	5	29	35	367	42	434
8	1176	4967	15	61	56	478	60	514
9	1108	4985	15	78	60	595	79	748
10	1165	4921	24	119	89	808	116	971
11	982	4726	22	158	113	975	141	1148
12	916	3863	39	156	111	1233	158	1453
13	477	3290	18	182	43	1215	46	1464
14	432	3047	10	169	39	1377	38	1562
15	370	2705	8	195	36	1422	36	1627
16	289	2359	17	184	27	1345	25	1432
17	231	2220	10	180	31	1334	38	1524
18	212	2463	13	225	31	1573	37	1657
19	193	2201	12	216	39	1494	24	1729
20	214	2333	10	260	31	1545	38	1693
21	199	2125	12	236	27	1341	36	1566
22	249	2128	20	267	38	1510	44	1589
23	405	2645	17	321	45	1757	103	1869
24	567	2930	37	338	88	1953	139	1997
25	839	3319	72	355	113	2047	203	2090
26	1072	3701	99	412	178	2230	284	2345
27	1412	3760	107	438	209	2365	339	2559
28	1432	1990	111	245	236	1247	324	1265
29	1355	1519	125	212	266	906	341	890
30	1259	1365	115	181	244	833	345	769
31	1020	1204	111	164	198	838	234	689
32	904	1348	113	177	190	895	249	843
33	758	1205	72	170	165	824	203	794
34	605	1207	64	178	128	775	148	759

NOTE: Because vaccination status was modeled as a time-varying covariate (see Materials and Methods), Veterans could contribute both vaccinated and unvaccinated time. Therefore, the total number of observations in the table does not represent the unique number of Veterans.

Supplementary Table 3D: RT-PCR Assays by Vaccination Status and Week, February 1 – October 1, 2021 (used for Figure 2D): Age >65 years

	Unvaccinated		Vaccinated					
			Janssen		Moderna		Pfizer-BioNTech	
Week	PCR+	PCR-	PCR+	PCR-	PCR+	PCR-	PCR+	PCR-
1	3107	8432	0	0	19	38	17	80
2	2053	8340	0	0	21	94	36	256
3	2315	10517	0	1	56	473	95	693
4	1905	9390	0	0	82	832	108	1195
5	1236	8659	0	0	78	1112	137	1599
6	1374	7528	0	2	139	1620	191	2103
7	1285	7163	3	39	203	2108	212	2379
8	1198	5657	5	56	219	2589	245	2634
9	1162	5375	17	74	268	3090	289	3055
10	1119	4691	20	101	286	3504	326	3241
11	960	4188	33	140	298	3727	354	3516
12	946	3466	47	157	336	3904	348	3671
13	504	3045	21	172	123	3901	136	3596
14	468	2856	11	173	108	3910	125	3763
15	397	2786	16	173	123	4005	99	3748
16	336	2396	10	194	104	3719	89	3574
17	262	2258	18	174	109	3675	86	3419
18	280	2535	12	224	94	4215	74	3994
19	242	2489	13	220	106	3977	86	3936
20	222	2392	15	249	82	4240	83	3786
21	208	2225	11	240	90	3945	90	3514
22	215	2116	10	228	94	3951	113	3526
23	342	2493	36	308	162	4749	218	4101
24	448	2544	33	336	235	4919	287	4294
25	626	2607	50	320	293	5008	402	4405
26	898	2645	86	342	474	5467	583	4809
27	1056	2665	94	363	575	5491	725	4982
28	1185	1576	126	235	636	3142	811	2585
29	1195	1282	114	181	755	2355	857	1835
30	1197	1120	119	168	681	2209	781	1721
31	938	1006	91	171	557	2087	618	1634
32	890	1134	94	195	604	2347	631	1852
33	707	1116	65	180	517	2279	485	1809
34	563	1147	52	184	462	2116	427	1709

NOTE: Because vaccination status was modeled as a time-varying covariate (see Materials and Methods), Veterans could contribute both vaccinated and unvaccinated time. Therefore, the total number of observations in the table does not represent the unique number of Veterans.

Supplementary Table 3E: Vital Status by Vaccination Status, RT-PCR Assay, and from RT-PCR Assay, Beginning February 1, 2021 (used for Figure 3A): Age <65 years

	Unvaccinated				Vaccinated			
	PCR-		PCR +		PCR-		PCR +	
Weeks from RT-PCR Assay	Deaths	Non-Deaths	Deaths	Non-Deaths	Deaths	Non-Deaths	Deaths	Non-Deaths
1	95	3932	74	1811	55	3747	11	677
2	164	3002	192	1731	92	2839	14	673
3	133	3285	173	2217	107	3068	9	853
4	120	2923	126	2497	91	2729	11	861
5	119	3297	88	3203	97	2990	17	1101
6	102	3622	43	3329	89	3315	4	1129
7	80	4648	27	3380	87	4457	7	1082
8	78	8459	23	3397	83	8480	5	1048
9	68	8036	15	2778	74	8075	3	937
10	64	6770	8	2157	66	7172	1	594
11	44	5949	8	1413	62	6678	1	404
12	50	5024	9	950	71	6266	3	258
13	43	3845	8	562	41	5276	4	149
14	33	3669	7	453	33	4941	0	124
15	29	4015	9	427	32	5466	2	122
16	25	3741	4	380	31	5513	0	117
17	21	4035	5	403	32	5674	1	106
18	21	3502	6	417	24	5048	3	114
19	12	3672	4	519	28	5028	1	105
20	20	3951	8	638	19	5668	1	114
21	18	4394	4	731	15	5747	1	122
22	18	4301	3	848	22	5737	0	139
23	20	4562	1	1404	14	5989	1	414
24	12	4845	4	1554	10	5869	1	362
25	12	4476	4	1634	14	5935	0	307
26	9	4255	3	1522	15	5556	0	198

NOTE: Numbers in table correspond to Kaplan-Meier curves shown in Figure 3 which were truncated at 26 weeks to avoid incomplete follow-up and sparse data. Therefore, the total number of observations in the table does not represent the total nested sample given in Materials and Methods.

Supplementary Table 3F: Vital Status by Vaccination Status, RT-PCR Assay, and Week from RT-PCR Assay, Beginning February 1, 2021 (used for Figure 3B): Age >65 years												
		Unvaccinated						Vaccinated				
		PCR-			PCR +			PCR-			PCR +	
Weeks from RT-PCR Assay		Deaths	Non-Deaths		Deaths	Non-Deaths		Deaths	Non-Deaths		Deaths	Non-Deaths
1		390	1542		348	708		459	5760		102	1241
2		698	1100		725	661		996	4267		237	1046
3		614	1102		670	821		959	4360		210	1300
4		530	953		401	852		813	3870		128	1210
5		436	1038		227	1059		756	4114		82	1508
6		355	1190		147	1032		665	4368		63	1648
7		301	1405		92	1011		564	5961		29	1488
8		276	2367		58	872		500	10820		19	1330
9		216	2280		63	729		449	10616		23	1084
10		192	2166		40	477		375	9777		17	692
11		180	2097		41	348		324	9563		13	517
12		163	2033		46	260		300	9179		16	395
13		114	1691		24	175		254	7676		17	203
14		113	1719		26	160		222	7698		13	170
15		87	1820		30	179		219	8341		12	166
16		67	1854		28	193		191	8207		7	197
17		65	1822		25	213		173	8563		8	167
18		55	1564		21	188		129	7364		8	195
19		56	1557		22	244		134	7657		12	186
20		50	1784		16	276		112	8222		10	220
21		52	1710		16	307		103	8228		8	230
22		44	1732		16	332		103	8179		10	259
23		33	1777		17	523		92	8422		6	682
24		28	1761		13	515		70	8391		2	646
25		28	1821		10	559		73	8179		2	585
26		15	1637		15	513		48	7925		5	541

NOTE: Numbers in table correspond to Kaplan-Meier curves shown in Figure 3 which were truncated at 26 weeks to avoid incomplete follow-up and sparse data. Therefore, the total number of observations in the table does not represent the total nested sample given in Materials and Methods.

Supplementary Table 3G: Vital Status by Vaccination Status, RT-PCR Assay, and Week from RT-PCR Assay, Beginning February 1, 2021 (used for Figure 3C): Charlson Comorbidity Index score <3													
		Unvaccinated						Vaccinated					
		PCR-			PCR +			PCR-			PCR +		
Weeks from RT-PCR Assay		Deaths	Non-Deaths		Deaths	Non-Deaths		Deaths	Non-Deaths		Deaths	Non-Deaths	
1		238	4881		209	2203		195	7051		31	1239	
2		382	3725		485	2080		384	5387		80	1133	
3		339	3953		462	2656		375	5588		80	1454	
4		294	3515		294	2945		292	4982		46	1362	
5		236	3961		178	3766		296	5372		27	1787	
6		202	4375		103	3902		261	5795		17	1841	
7		179	5423		60	3897		219	7462		10	1723	
8		164	9542		34	3792		196	13031		5	1593	
9		120	9084		35	3153		174	12549		8	1390	
10		122	7782		18	2374		148	11097		9	866	
11		105	6890		23	1580		123	10559		6	630	
12		106	5954		18	1078		114	9972		2	423	
13		63	4620		10	651		102	8305		4	214	
14		69	4401		15	514		95	8069		5	189	
15		53	4856		18	524		87	8871		3	185	
16		49	4602		10	482		79	8953		1	208	
17		44	4854		10	513		76	9263		2	181	
18		41	4199		7	509		60	8090		2	192	
19		38	4388		11	651		69	8288		3	186	
20		32	4778		10	777		44	9218		1	196	
21		38	5230		8	878		42	9298		3	198	
22		24	5155		8	1025		53	9298		5	248	
23		22	5385		8	1666		37	9615		1	637	
24		23	5656		6	1769		33	9503		0	605	
25		21	5339		7	1879		27	9610		1	504	
26		14	5047		7	1747		21	9078		1	410	

NOTE: Numbers in table correspond to Kaplan-Meier curves shown in Figure 3 which were truncated at 26 weeks to avoid incomplete follow-up and sparse data. Therefore, the total number of observations in the table does not represent the total nested sample given in Materials and Methods.

Supplementary Table 3H: Vital Status by Vaccination Status, RT-PCR Assay, and Week from RT-PCR Assay, Beginning February 1, 2021 (used for Figure 3D): Charlson Comorbidity Index score ≥3

		Unvaccinated						Vaccinated				
		PCR-			PCR +			PCR-			PCR +	
Weeks from RT-PCR Assay		Deaths	Non-Deaths		Deaths	Non-Deaths		Deaths	Non-Deaths		Deaths	Non-Deaths
1		247	593		213	316		319	2456		82	679
2		480	377		432	312		704	1719		171	586
3		408	434		381	382		691	1840		139	699
4		356	361		233	404		612	1617		93	709
5		319	374		137	496		557	1732		72	822
6		255	437		87	459		493	1888		50	936
7		202	630		59	494		432	2956		26	847
8		190	1284		47	477		387	6269		19	785
9		164	1232		43	354		349	6142		18	631
10		134	1154		30	260		293	5852		9	420
11		119	1156		26	181		263	5682		8	291
12		107	1103		37	132		257	5473		17	230
13		94	916		22	86		193	4647		17	138
14		77	987		18	99		160	4570		8	105
15		63	979		21	82		164	4936		11	103
16		43	993		22	91		143	4767		6	106
17		42	1003		20	103		129	4974		7	92
18		35	867		20	96		93	4322		9	117
19		30	841		15	112		93	4397		10	105
20		38	957		14	137		87	4672		10	138
21		32	874		12	160		76	4677		6	154
22		38	878		11	155		72	4618		5	150
23		31	954		10	261		69	4796		6	459
24		17	950		11	300		47	4757		3	403
25		19	958		7	314		60	4504		1	388
26		10	845		11	288		42	4403		4	329

NOTE: Numbers in table correspond to Kaplan-Meier curves shown in Figure 3 which were truncated at 26 weeks to avoid incomplete follow-up and sparse data. Therefore, the total number of observations in the table does not represent the total nested sample given in Materials and Methods.

Supplementary Table 3I: Vital Status by Vaccination Status, RT-PCR Assay, and Week from RT-PCR Assay, Beginning July 1, 2021 (used for Figure S1A): Age <65 years

	Unvaccinated				Vaccinated			
	PCR-		PCR +		PCR-		PCR +	
Weeks from RT-PCR Assay	Deaths	Non-Deaths	Deaths	Non-Deaths	Deaths	Non-Deaths	Deaths	Non-Deaths
1	30	3932	35	1811	19	3747	10	677
2	33	3002	127	1731	42	2839	11	673
3	17	3285	104	2217	32	3068	7	853
4	27	2923	85	2497	34	2729	6	861
5	19	3297	44	3203	24	2990	13	1101
6	19	3622	24	3329	33	3315	1	1129
7	16	4648	9	3380	17	4457	5	1082
8	12	8459	5	3397	22	8480	2	1048

NOTE: Numbers in table correspond to Kaplan-Meier curves shown in Supplementary Figure 1 which included RT-PCR results beginning July 1, 2021 onward to examine the time period corresponding to the dominance of the Delta variant. Owing to the shorter follow-up period these curves were truncated at 8 weeks to avoid incomplete follow-up and sparse data.

Supplementary Table 3J: Vital Status by Vaccination Status, RT-PCR Assay, and Week from RT-PCR Assay, Beginning July 1, 2021 (used for Figure S1B): Age ≥65 years

	Unvaccinated				Vaccinated			
	PCR-		PCR +		PCR-		PCR +	
Weeks from RT-PCR Assay	Deaths	Non-Deaths	Deaths	Non-Deaths	Deaths	Non-Deaths	Deaths	Non-Deaths
1	79	1542	131	708	178	5760	64	1241
2	133	1100	345	661	383	4267	185	1046
3	86	1102	323	821	333	4360	149	1300
4	105	953	177	852	255	3870	92	1210
5	75	1038	83	1059	230	4114	51	1508
6	55	1190	38	1032	182	4368	27	1648
7	58	1405	25	1011	158	5961	15	1488
8	40	2367	8	872	119	10820	10	1330

NOTE: Numbers in table correspond to Kaplan-Meier curves shown in Supplementary Figure 1 which included RT-PCR results beginning July 1, 2021 onward to examine the time period corresponding to the dominance of the Delta variant. Owing to the shorter follow-up period these curves were truncated at 8 weeks to avoid incomplete follow-up and sparse data.

Supplementary Figure 1. Kaplan-Meier curves illustrating cumulative risk of death due to any cause by vaccination status and RT-PCR assay for the period beginning July 1, 2021, corresponding to the emergence and dominance of the Delta variant in the U.S.: A) age <65 years; B) age ≥65 years

A. Age <65 years

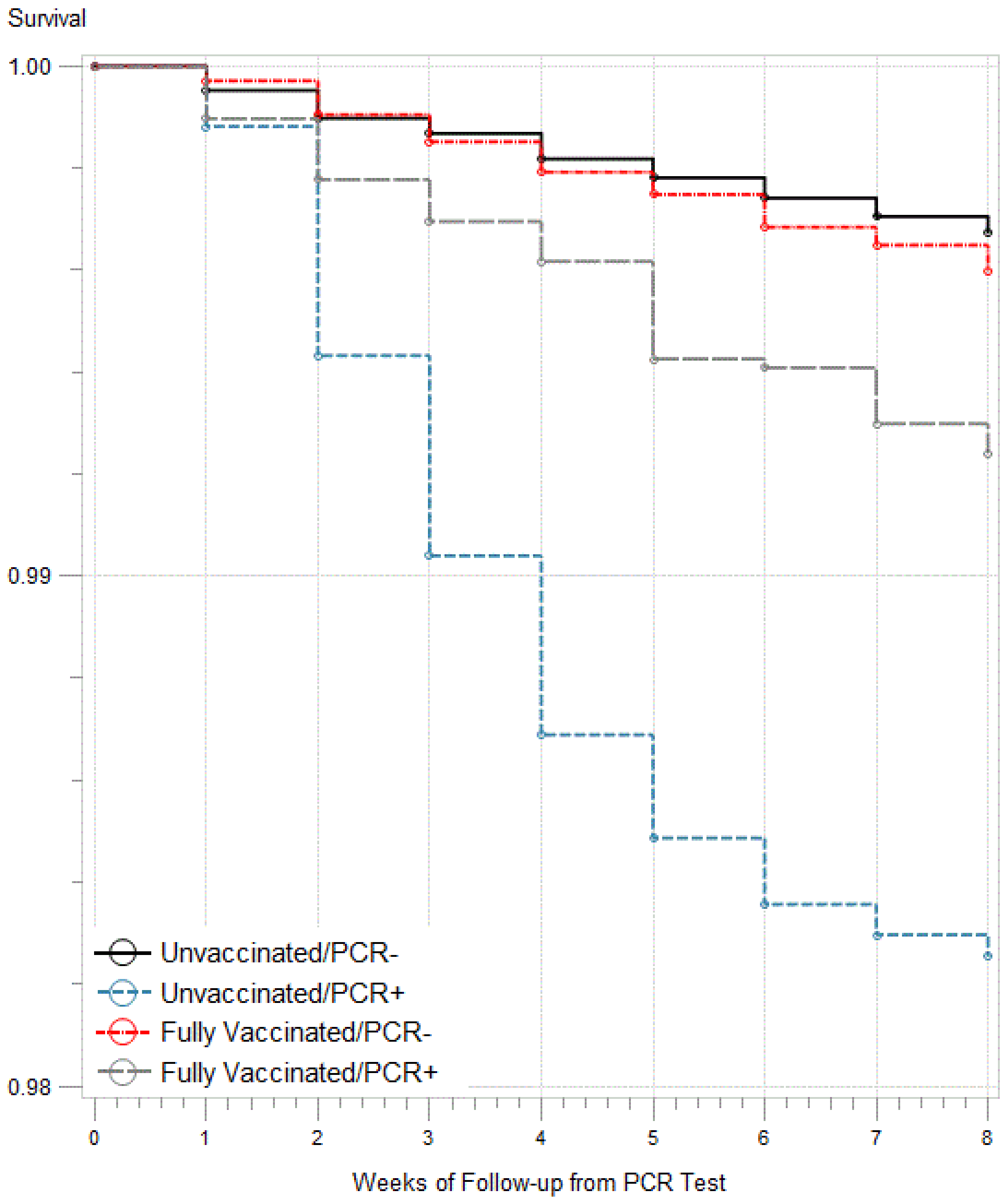

B. Age ≥65 years

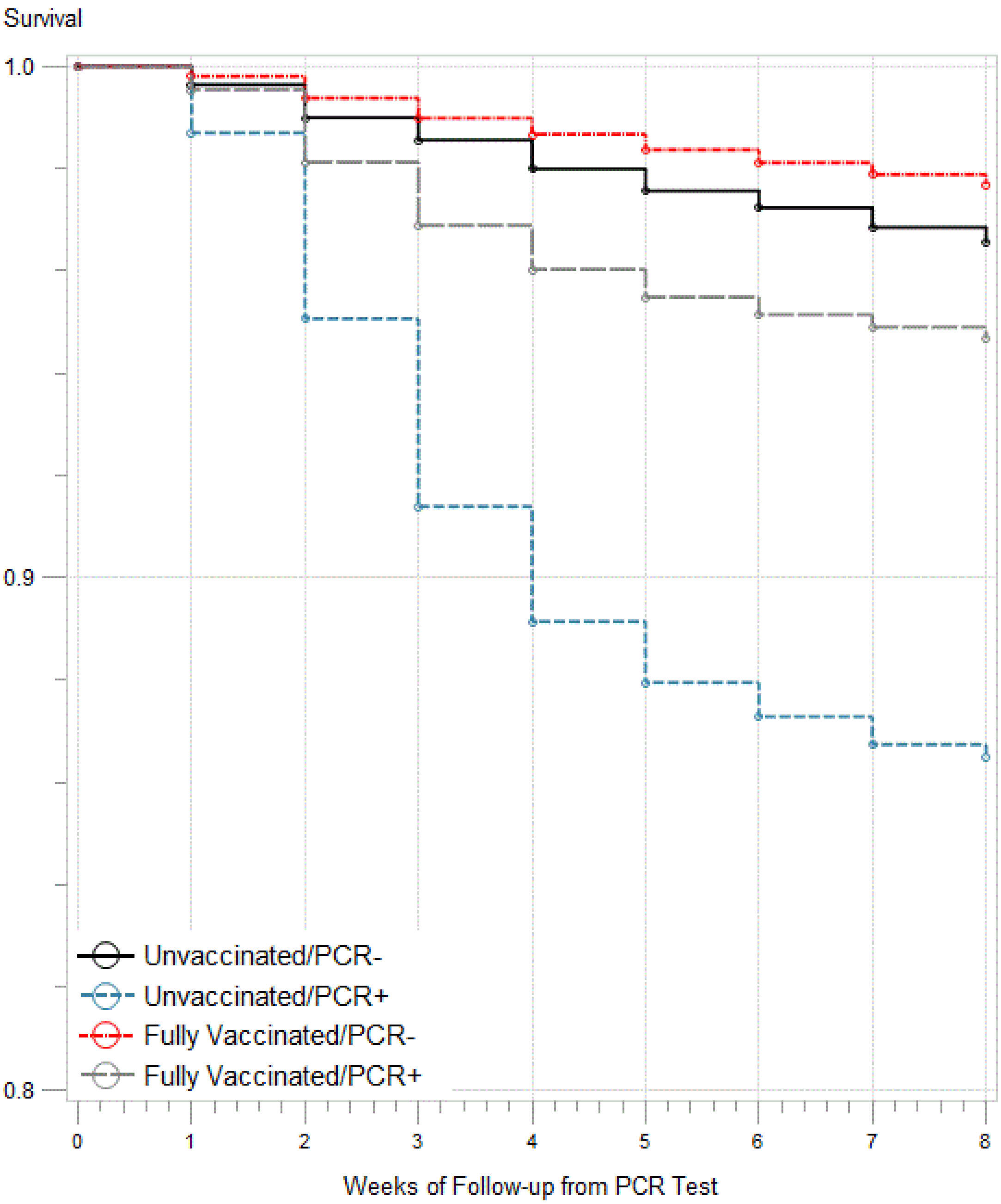

Supplement: Supplementary file 2 — Material and Methods Fig. S1 Tables S1 to S3 [file science.abm0620_sm.pdf]
